# ﻿Desert diversification: revision of *Agroecotettix* Bruner, 1908 (Orthoptera, Acrididae, Melanoplinae) with descriptions of sixteen new species from the United States and Mexico

**DOI:** 10.3897/zookeys.1218.133703

**Published:** 2024-11-21

**Authors:** JoVonn G. Hill

**Affiliations:** 1 Mississippi Entomological Museum, Department of Molecular Biology, Biochemistry, Entomology, and Plant Pathology, Mississippi State University, Starkville, USA Mississippi State University Starkville United States of America

**Keywords:** Aridland scrub jumpers, Big Bend National Park, biodiversity, Chihuahuan Desert, Sierra Madre Oriental, Trans Pecos region

## Abstract

In this study, a morphological revision was conducted of *Agroecotettix* Bruner, a group of grasshoppers inhabiting open xeric desert scrub, shrublands, and plains, spanning central Texas to central Mexico. The genus was originally described by Bruner in 1908, with two taxa added by Hebard in 1922. *Agroecotettix* has remained unrevised despite numerous collections. This exploration, spurred by a novel discovery of significant male genitalia variation in *Agroecotettixaristusaristus*, suggests undescribed species. Through morphological specimen comparisons, sixteen new species are described from biologically rich regions of the South Texas Plains, Chihuahuan Desert, and Sierra Madre Oriental. The new taxa described here are *A.silverheelsi***sp. nov.**, *A.xiphophorus***sp. nov.**, *A.glochinos***sp. nov.**, *A.texmex***sp. nov.**, *A.cumbres***sp. nov.**, *A.burtoni***sp. nov.**, *A.moorei***sp. nov.**, *A.chiantiensis***sp. nov.**, *A.dorni***sp. nov.**, *A.chisosensis***sp. nov.**, *A.turneri***sp. nov.**, *A.quitmanensis***sp. nov.**, *A.vaquero***sp. nov.**, *A.forcipatus***sp. nov.**, *A.idic***sp. nov.**, and *A.kahloae***sp. nov.** This discovery sheds light on desert biodiversity and hints at a Pleistocene radiation akin to other melanoplines, urging further exploration to enrich our understanding of this fascinating lineage and unravel the biogeographic history within these arid landscapes.

## ﻿Introduction

*Agroecotettix* Bruner, 1908 are commonly found in open shrublands, plains, and xeric desert scrub from central Texas to southeastern New Mexico, south to central Mexico. Bruner (1908) established the genus with the description of *Agroecotettixmodestus* based on a female collected from Ciudad Lerdo, Coahuila, Mexico. Hebard (1922) added *Agroecotettixaristusaristus* and *Agroecotettixaristuscrypsidomus* from Uvalde and Marathon, Texas, USA respectively, resulting in three described taxa.

Despite the passage of time since Hebard’s work, species hypotheses in *Agroecotettix* have not been tested. Numerous collections have provided a wealth of material for study over the years, including those of Dr. Ted Cohn from Mexico and recent efforts by the Mississippi Entomological Museum from Texas. During examination of specimens collected from Texas in 2020, a notable variation in the male genitalia of *A.aristus*, was suggestive that an undescribed species was present. Characters of the male genitalia have long been used for species hypotheses of Melanoplinae, and those hypotheses have withstood testing by molecular analyses (Hubbell 1932; Otte 2012; Hill 2015; Woller 2017; Huang et al. 2020).

Upon gathering numerous specimens of *Agroecotettix*, it became evident that the genus is distributed across several biologically diverse regions, including the Edwards Plateau, the South Texas Plains, the Chihuahuan Desert, and the Sierra Madre Oriental. Given the recent description of cryptic diversity in other melanopline grasshoppers in these areas (Otte 2012; Barrientos-Lozano et al. 2013a; Otte 2019; Hill 2023), it is likely that further explorations will unveil additional species of *Agroecotettix*.

## ﻿Materials and methods

Most specimens examined in this study were borrowed from the University of Michigan Museum of Zoology (**UMMZ**) and the Academy of Natural Sciences of Drexel University (**ANSP**). Other specimens were collected by staff of the Mississippi Entomological Museum (**MEM**) during the summers of 2018–2023. Specimens were obtained by capturing them with a standard insect net. The captured individuals were placed into a jar containing potassium cyanide, for pinning, or 100% ethanol for molecular work. Specimens from New Mexico were borrowed from Brigham Young Arthropod Museum (**BYUC**) and the University of Kansas Natural History Museum (**SEMC**). All type specimens of newly described species are deposited in the MEM. Nomenclature follows Cigliano et al. (2024). Specimens collected by the MEM have been databased in the Symbiota Collection of Arthropods Network.

Internal male genitalia, which are typically concealed within the terminalia, were either exposed upon pinning fresh specimens, or the specimen was relaxed by soaking in warm water, then the genital mass was either extruded or dissected and examined in a manner similar to Gurney and Brooks 1959. Terminology for external morphology and male genitalia follows Carbonell (2007) and Eades (2000). Habitus and internal genitalia images were produced using a Leica DFC 495 digital camera mounted on a Leica Z16 microscope with motorized z-stepping. Image stacks were merged using Leica Application Suite V 4.1.0 with the Montage Module. Images were edited using Adobe Photoshop CS6 software. A green label stating “Measured by JGH” was added to the specimens measured in this study. Measurements were made with a Leica MZ 12.5 stereomicroscope with a reticule in the following ways:

- Body length — Dorsally from the fastigium verticis to the distal end of the genicular lobe of the hind femur in a parallel plane with the abdomen
- Pronotum length — Dorsally, along the median carina
- Male cercus length — Laterally, maximum measurement of the left cercus
- Male cercus basal width — Laterally, along the point of attachment from the dorsal to ventral margin
- Male mid cercus width — Laterally, at the mid-length of the left cercus
- Male cercus ventral branch length — Laterally, from beginning of the fork to the apex
- Male cercus ventral branch apex width — Laterally, along the distal end
- Male cercus dorsal branch length — Laterally, from beginning of the fork to the apex
- Male cercus dorsal branch apex width — Laterally, along the distal end
- Female dorsal ovipositor valve — Laterally, from the base to the apex
- Female ventral ovipositor valve — Laterally, from the base to the apex

## ﻿Taxonomic account

### ﻿ Agroecotettix

Taxon classificationAnimaliaOrthopteraAcrididae

Bruner 1908

9D41E3EB-924B-5A4E-8261-03D610239B15

#### Diagnosis.

**Figure 1.**
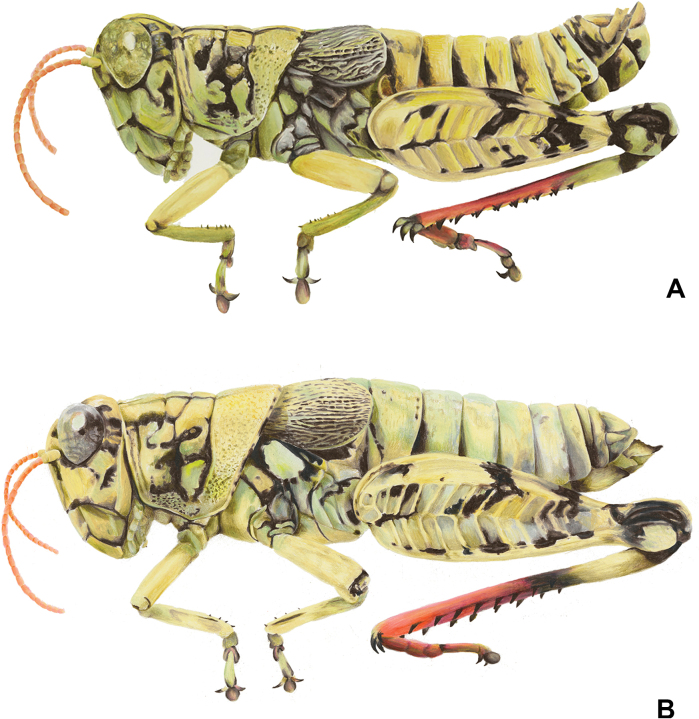
Habitus illustrations of *Agroecotettixaristus***A** male **B** female. Created by Ashley Baker.

**Figure 2.**
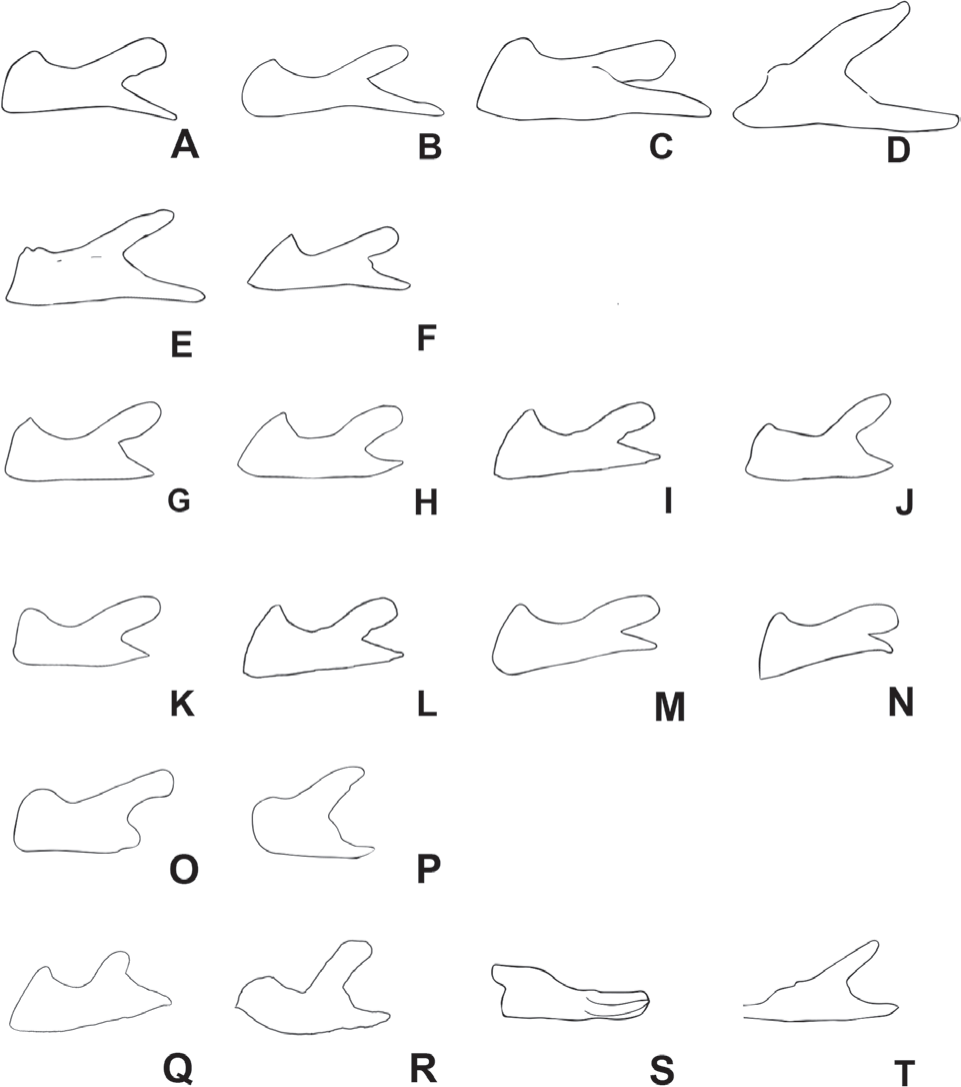
*Agroecotettix* cerci in lateral view unless noted otherwise **A***A.aristus***B***A.silverheelsi***C***A.xiphophorus***D***A.xiphophorus* ventral view **E***A.texmex***F***A.cumbres***G***A.crypsidomus***H***A.chisosensis***I***A.dorni***J***A.burtoni***K***A.turneri***L***A.quitmanensis***M***A.moorei***N***A.chiantiensis***O***A.vaquero***P***A.forcipatus***Q***A.kahloae***R***A kahloae* ventral view **S***A.idic* lateral view **T***A.idic* dorsal.

**Figure 3.**
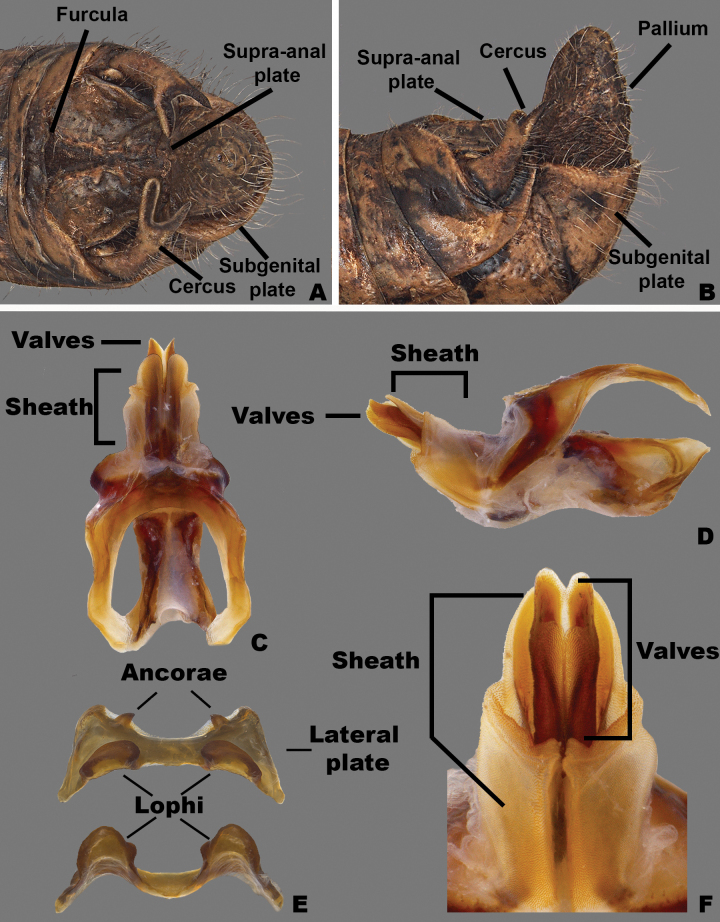
Morphology of the male terminalia and phallic complex of *Agroecotettix* used in this work **A** dorsal view of male terminalia **B** Lateral view of the male terminalia **C** dorsal view of the phallic complex **D** lateral view of the phallic complex **E** epiphallus dorsal and caudal views **F** caudal view of the aedeagus.

**Figure 4.**
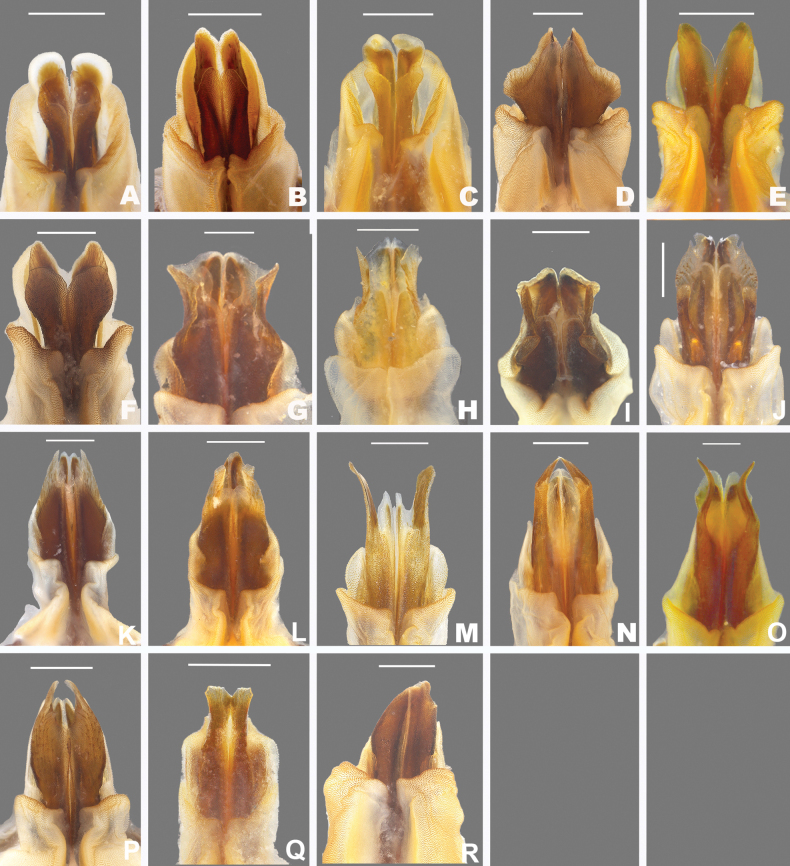
Caudal view of the aedeagus of *Agroecotettix***A**. *A.silverheelsi***B***A.aristus***C***A.xiphophorus***D***A.glochinos***E***A.texmex***F***A.cumbres***G***A.crypsidomus***H***A.burtoni***I***A.moorei***J***A.chiantiensis***K***A.forcipatus***L***A.quitmanensis***M***A.dorni***N***A.chisosensis***O***A.turneri***P***A.vaquero***Q***A.idic***R***A.kahloae*. Scale bars: 1.0 mm.

**Figure 5.**
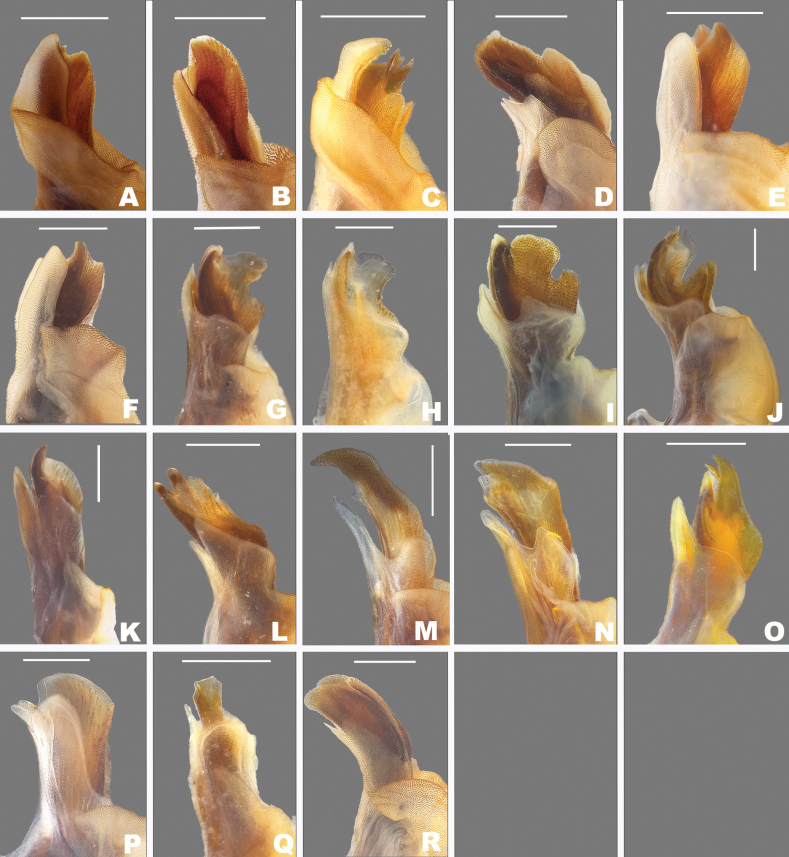
Lateral view of the aedeagus of *Agroecotettix***A***A.silverheelsi***B***A.aristus***C***A.xiphophorus***D***A.glochinos***E***A.texmex***F***A.cumbres***G***A.crypsidomus***H***A.burtoni***I***A.moorei***J***A.chiantiensis***K***A.forcipatus***L***A.quitmanensis***M***A.dorni***N***A.chisosensis***O***A.turneri***P***A.vaquero***Q***A.idic***R***A.kahloae*.

A genus of medium-sized (18–31.1 mm) brachypterous grasshoppers (Fig. 1). Head large and as broad or slightly broader than the prozona; vertex between the eyes slightly wider than the basal antennomere; fastigium broadly rounded being more pronounced dorsally than ventrally, with a shallow medial depression throughout. Eyes somewhat prominent, especially in males. Three ocelli present. Antennae filiform, usually with 23 flagellomeres, but occasionally 24 or 25; nearly cylindrical, but slightly flattened dorso-ventrally; equal in width throughout, except two basal articles. ***Thorax*** with prosternal spine well-developed; subquadrate basally and acutely pointed distally. Pronotum slightly convex, anterior and caudal margins sub-truncate, lateral margins sub parallel. Prozona mostly smooth, with light punctation along the apical margin, then smooth throughout; lateral lobes quadrate (more so in females) with parallel lateral margins and the ventral margin sloping ventrally caudally. Metazona punctate throughout, with humeral margins rounded, slightly diverging posteriorly in dorsal view. Median carina low, but distinct throughout, except where the sulci cross it. Anterior, median, and posterior sulci are apparent due to their black coloration, and all dissect the median carina and nearly reach the ventral margin of the lateral lobes. Lateral pronotal margins broadly rounded on the prozona and slightly angular on the metazona. Interspace between mesosternal lobes nearly twice as long as broad. Tegmina broadly oval; dorsal margins only slightly separated dorsally; strongly veined; extending little past the anterior margin of the first abdominal tergite. Pro and meso thoracic legs robust, inflated, and bowed. Hind femur enlarged with basal end bi-lobed. Hind tibia with 11 or 12 pairs of spines, but typically 11. Tympanum present under tegmina, appearing as an opaque whitish disk. Abdomen cylindrical with distal portion distinctly, but not greatly enlarged. ***Terminalia of male*** with bifurcate cerci (Figs 2A–T, 3A, B), longer than wide, but the length and angle of the branches varies between species, the dorsal branch is rounded distally and flattened ventrally, the ventral branch is produced as straight and slender spike. Subgenital plate with a low but even margin. Furcula (Fig. 3A) typically broadly rounded protuberances, projecting slightly beyond the end of the segment from which they originate; well separated. Supra-anal plate (Fig. 3A, B) of male broadly triangular, length equal to the width of the base, with the median groove anteriorly distinct with elevated sides that fade caudally; with a small median tubercule lateral to the groove. Pallium evident, sometimes prominent (Fig. 7B) and covering the dorsally projecting internal genitalia. ***Phallic structures*** (Fig. 3C–F). The valves of the aedeagus are quite variable between species, but in *Agroecotettix* the dorsal and ventral valves appear to be fused into a single structure that when paired bilaterally form a central channel. In the *aristus* group, the sheath of the aedeagus is produced as thickened, fleshy projections on the dorsal side of the valves (Figs 4A–F, 5A–F). The valves in the *aristus* group are entire in lateral profile (Fig. 5A–F). In the *crypsidomus* group, the sheath of the aedeagus is produced as thin projections on the dorsal side of the valves (Figs 4G–R, 5G–R). The valves of most species in the *crypsidomus* group are often lobate or undulate in lateral profile, though some are entire (Fig. 5G–R). The epiphallus is of the typical melanoploid shape, having lophi, ancorae, and an undivided bridge, but more precisely, members of *Agroecotettix* have a concave bridge, broadly rounded lophi, convexly curved lateral plates that are subdeltate in shape with a rounded anterior lobe and an acuminate caudal tip, and ancorae that are triangular, often tapering to a point (Fig. 3C, D).

**Figure 6.**
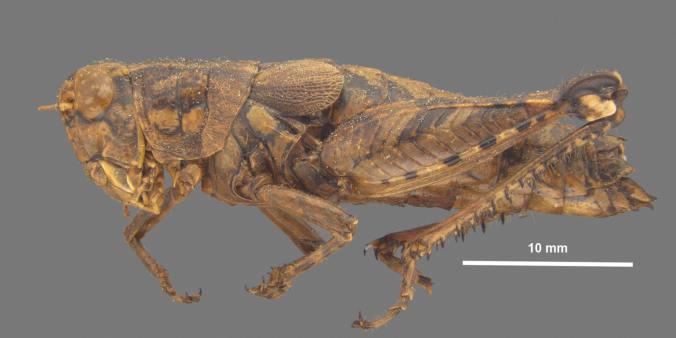
Habitus of type specimen of *Agroecotettixmodestus*.

Females are similar to the males, but differ in being larger, more robust, with proportionately broader tegmina, and in the shape of the terminalia (Figs 1B, 6, 15C, E, 16D, 17E, F, 18G, H, 19D, 21C). ***Terminalia of female*** with triangular cerci and ovipositor valves that are subequal in length. The dorsal valves with their dorsum being nodose to slightly serrate proximally and concave and upcurving to a tip distally. The ventral valves with their ventral margins straight basally and then arching distally (Fig. 6).

**Figure 7.**
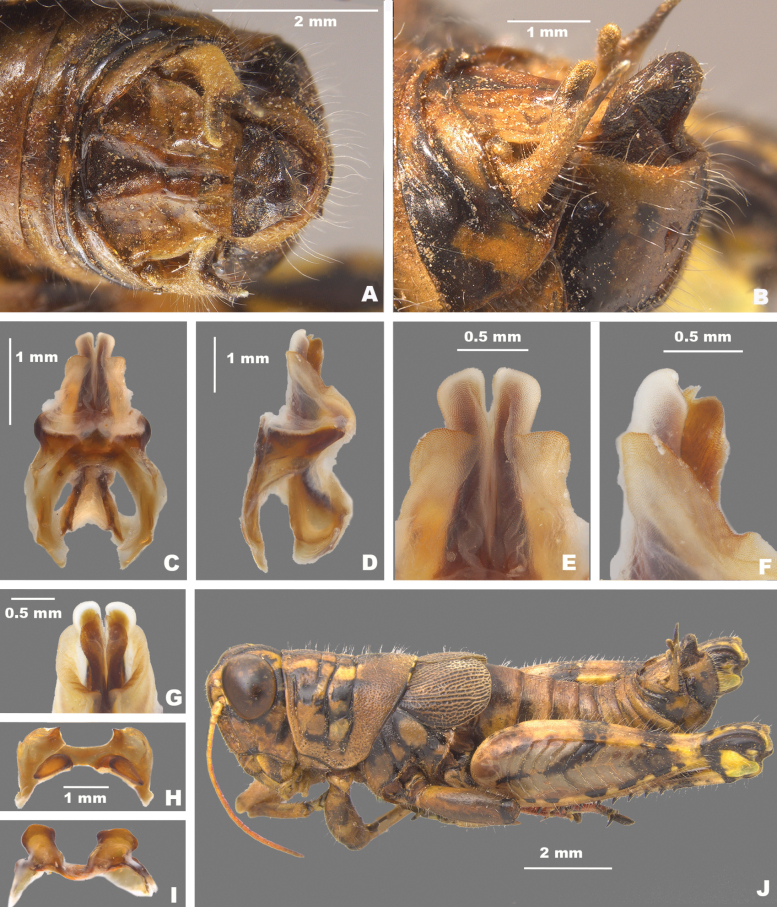
*Agroecotettixsilverheelsi***A** dorsal view of male terminalia **B** lateral view of male terminalia **C** dorsal view of phallic complex **D** lateral view of phallic complex **E** dorsal view of aedeagus **F** lateral view of aedeagus **G** caudal view of the aedeagus **H** dorsal view of epiphallus **I** caudal view of epiphallus **J** habitus.

#### Coloration.

Ochraceous (brownish yellow) overall, with individual variation that can be either a tawny or cinereous hue (see Figs 26–35), with a conspicuous round, lighter tawny spot laterad on the metathorax near the bast of the tegmina. Head with black markings, including a band along the dorsum, flecks on the genae and a post-ocular stripe. Pronotum with the post-ocular stripe continuing onto the prozona and mesosoma and then disappearing on the metazona; median carinae and sulci black. The wings are dark brown with a network of ochraceous veins. Abdomen with proximal tergites suffused with black spots. Ventral surface of the body pale with black sutures between the sternites. Male subgenital plate with a medial black spot. The fore and middle legs unmarked. The hind femur with two thick transverse bands laterally and suffused with black dorsally; genicular area black with ochraceous lobes; inner face bright yellow, and coral-red ventrally and crossed by black bands distally. Hind tibia with the first third ochraceous proximally, then with a ring of black, remaining two-thirds bright coral red with black tipped spines.

#### Etymology.

*Agro* Latin = open country, *eco* Greek home, *tettix* Greek grasshopper.

#### Suggested common name.

Aridland scrub jumpers. In reference to the arid habitat and plant community in which these grasshoppers are found.

*Agroecotettix* superficially resemble *Phaulotettix*, but can be differentiated as follows:

*Agroecotettix*

1. Tegmina broad and oval; attingent, nearly touching dorsally
2. Metathorax with a pale-colored spot
3. Cerci bifurcated
4. Hind tibia gray or yellow proximately, turning bright red distally
5. Inside of hind femur red
6. Furculae short and broad, and well separated from each other

*Phaulotettix*

1. Tegmina linear; their dorsal margins widely separated dorsally
2. Metathorax with a pale-colored stripe
3. Cerci falcate, simple
4. Hind tibia blue proximately, turning red distally
5. Inside of hind femur not red
6. Furculae short, obvious, linear; attingent or touching

##### ﻿*Key to*Agroecotettix

**Table:** 

1	Male cerci with ventral branch longer than dorsal branch as in Fig. 2A–F; sheath of aedeagus produced as thickened fleshy lobes dorsal to the valves as in Figs 4A–F, 5A–F	***A.aristus* group 3**
–	Male cerci with ventral branch equal, subequal, or shorter than the dorsal branch as in Fig. 2G–T; sheath of aedeagus not produced as thickened fleshy lobes dorsal to the valves (Figs 4G–R, 5G–R)	**8**
2	Occurring north of the Rio Grande River in the United States	**3**
–	Occurring south of the Rio Grande River in Mexico	**5**
3	Valves of aedeagus shorter than the sheath in lateral view; and with broadly rounded apices (Figs 4B, 5B); found in the South Texas Plains (Fig. 25A)	***A.silverheelsi* sp. nov.**
–	Valves of aedeagus longer, extending well beyond the sheath in lateral view (Fig. 4A)	**4**
4	In caudal view, the valves of the aedeagus are relatively narrower and forming parallel dorsal and ventral arches that are narrowly rounded at their apices as in Fig. 4B; in lateral view; the distal edge of the valves are broadly rounded as in Fig. 5B; found across central west Texas to southern New Mexico (Fig. 25A)	***A.aristus* Hebard**
–	In caudal view, the valves of the aedeagus are relatively more wider and form distally diverging arches that are more broadly rounded at their apices as in Fig. 4E; In lateral view, the distal edge of the valves forming an acute point dorsally, but with a broadly rounded ventral edge (Fig. 5E); found in extreme southern Texas in the vicinity of Jim Hogg County (Fig. 25A)	***A.texmex* sp. nov.** (in part)
5	Male cerci with branches widely separated and with the ventral branch much longer than the dorsal branch as in Fig. 2C, D; valves of the aedeagus with their dorsal margin somewhat bilobed and the ventral margin broadly rounded and with their distal apices diverging laterally as in Figs 4C, 5C; found in west-central Nuevo Leòn, Mexico (Fig. 25A, C)	***A.xiphophorus* sp. nov.**
–	Male cerci with branches not widely separated and with the ventral branch only slightly longer than the dorsal branch as in Fig. 2E, F; valves of the aedeagus with their dorsal margin not bilobed	**6**
6	Valves of the aedeagus with their lateral margins greatly expanded centrally and with the dorsal apices forming acute parallel points and the ventral apices forming rounded parallel arches in caudal view as in Fig. 4D; in lateral view the valves are directed apically (Fig. 5D); found in the vicinity of Galeana, MX (Fig. 25A, C)	***A.glochinos* sp. nov.**
–	Valves of the aedeagus with their apices diverging distally in caudal view as in Fig. 4D, E; in lateral view the valves are directed more caudally as in Fig. 5D–F	**7**
7	In caudal view, the valves of the aedeagus relatively narrower (Fig. 4E); in lateral view, the ventral edge of the valves are broadly rounded at their apices (Fig. 5E); found in the vicinity northern Nuevo León, Mexico (Fig. 25A)	***A.texmex* sp. nov.** (in part)
–	In caudal view, the valves of the aedeagus are very broad (Fig. 4F); and are more broadly rounded at their apices (Fig. 4F) and in lateral view with their distal edge forming an acute point dorsally and the ventral edge truncated (Fig. 5F); found in the vicinity of Galeana, Mexico (Figs 25A, 26B)	***A.cumbres* sp. nov.**
8	In lateral view, the valves of the aedeagus are lobate as in Fig. 5G–J	***A.crypsidomus* group 9**
–	In lateral view, the valves of the aedeagus are falcate or quadrate laterally as in Fig. 5K–R	**13**
9	In lateral view, the distal lobes of the aedeagus valves are more widely incised and the basal lobes are small or absent as in as in Fig. 5G, H, and in caudal view the dorsal valve is deeply undulate as in Fig. 4G, H	**11**
10	In lateral view, the apical lobes of the aedeagus valves are narrowly, but deeply incised resulting in the basal lobe being more pronounced and obvious as in Fig. 5I, J	**12**
11	In lateral view, basal lobe of aedeagus valves extending much beyond the sheath (Fig. 4G); dorsal lobes projected laterally in caudal view (Fig. 5G); Marathon, Texas (Figs 25, 26A)	***A.crypsidomus* Hebard**
–	In lateral view, basal lobe of aedeagus valves not extending much beyond the sheath (Fig. 5H); dorsal lobe almost vertical or curving medially (Fig. 5H); Big Bend region of Texas (Figs 25, 26A)	***A.burtoni* sp. nov.**
12	In lateral view, the valves of the aedeagus are shallowly incised with a broad distal lobe that is truncated apically, and the basal lobe is shorter (Fig. 5I); in caudal view the valves of the aedeagus are concave as in Fig. 4I; Found in the vicinity of Sanderson, Texas (Figs 25, 26A)	***A.moorei* sp. nov.**
–	In lateral view, the valves of the aedeagus are deeply incised with a narrower and slightly acute distal lobe, and a longer basal lobe as in Fig. 5J; in caudal view the valves of the aedeagus are convex as in Fig. 4J; found in the Chianti Mountains of southern Texas (Figs 25, 26A)	***A.chiantiensis* sp. nov.**
13	Occurring north of the Rio Grande River in the United States	**14**
–	Occurring south of the Rio Grande River in Mexico	**17**
14	In lateral view, the apices of the valves of the aedeagus point caudally as in Fig. 5M, found in the vicinity of western Brewster County, Texas (Figs 25, 26A)	***A.turneri* sp. nov.**
–	In lateral view, the apices of the valves of the aedeagus curve apically as is Fig. 5L–O	**15**
15	In lateral view, the apical edge of the valves of the aedeagus are thicker finger-like projections as in Fig. 5L; found in the vicinity of the Quitman Mountains in Hudspeth County, Texas (Figs 25, 26A)	***A.quitmanensis* sp. nov.**
–	In lateral view, the apical edge of the valves of the aedeagus are thin blade-like projections as in Fig. 5M, N)	**16**
16	In lateral view, the valves of aedeagus thinly falcate, long and sword-like as in Figs 5M, and in caudal view the lateral margins extend well beyond the rest of the valves and their apical margins are slightly curved distally as in 4M; found in the southern Big Bend region of Texas (Figs 25, 26A, B)	***A.dorni* sp. nov.**
–	In lateral view, the valves of aedeagus broad as in Fig. 5N, and in caudal view the lateral margins do not extend well beyond the rest of the valves and their apical margins are curved medially as in Fig. 4N; endemic to the Chisos Mountains of the Big Bend region of Texas (Figs 25, 26A)	***A.chisosensis* sp. nov.**
17	Male cerci with the ventral branch reduced and rounded as in Fig. 2O; in lateral view, the sheath of the aedeagus is well developed and expanded laterally around the valves; in lateral view, the aedeagus valves are wide with their apices broadly curved (Fig. 5P); in caudal view the valves or greatly narrowed in their apical third as in Fig. 4P; found in northern Coahuila, Mexico (Fig. 25)	***A.vaquero* sp. nov.**
–	Male cerci longer with both dorsal and ventral branches well produced	**18**
18	Male cerci short and not curved medially as in Fig. 2P; in lateral view, the valves of the aedeagus are acutely pointed apically and are greatly widened in their lower half; in caudal view the apical margins of the valves are slightly curved distally as in Fig. 4O; found southern Coahuila, Mexico in the vicinity of the Sierra de la Gloria (Fig. 25)	***A.forcipatus* sp. nov.**
–	Male cerci longer (Fig. 2Q–T); dorsal and lower branches subequal in length	**19**
19	Male cercus gently curved medially (Fig. 2Q, R); in lateral view the valves of the aedeagus are narrowly quadrate with the distal apices truncate apically as in Figs 4Q, 5Q; in caudal view the valves are also quadrate with the apical margin truncate as in Figs 4R, 5R; found in the vicinity of Saltillo, Mexico (Figs 25, 26B)	***A.idic* sp. nov.**
–	Male cercus strongly curved medially (Fig. 2S, T); in lateral view, the valves of the aedeagus are broad and arching with the distal apices rounded (Fig. 5R); in caudal view, valves acuminate; Arteaga, Mexico (Figs 25, 26B)	***A.kahloae* sp. nov.**

##### ﻿*Agroecotettix* species checklist

###### ﻿*Incertae sedis*

1. *Agroecotettixmodestus* Brunner, 1908, stat. nov. — Figs 6, 25

###### ﻿*A.aristus* group

2. *Agroecotettixsilverheelsi* sp. nov. — Figs 2B, 4A, 5A, 7A–J, 23A–C, 25, 26A

3. *Agroecotettixaristus* Hebard, 1922, stat. nov. — Figs 2A, 4B, 5B, 8A–J, 25, 26A, 28A–E, 29A–E

4. *Agroecotettixxiphophorus* sp. nov. — Figs 2C, D, 4C, 5C, 9A–J, 25, 26B

5. *Agroecotettixglochinos* sp. nov. — Figs 4D, 5D, 10A–J, 25, 26B

6. *Agroecotettixtexmex* sp. nov. — Figs 2E, 4E, 5E, 11A–J, 25, 26B

7. *Agroecotettixcumbres* sp. nov. — Figs 2F, 4F, 5F, 12A–J, 25, 26B

*A.crypsidomus* group

8. *Agroecotettixcrypsidomus* Hebard, 1922, stat. nov. — Figs 2G, 4G, 5G, 13A–J, 25, 26A, 31A–F

9. *Agroecotettixburtoni* sp. nov. — Figs 2J, 4H, 5H, 14A–J, 25, 26A, 31A–D

10. *Agroecotettixmoorei* sp. nov. — Figs 2M, 4I, 5I, 15A–J, 25, 26A, 32A–D

11. *Agroecotettixchiantiensis* sp. nov. — Figs 2N, 4J, 5J, 16A–J, 25, 26A, 33A–C

12. *Agroecotettixdorni* sp. nov. — Figs 2I, 4M, 5M, 17A–J, 25, 26A, 34A–D

13. *Agroecotettixchisosensis* sp. nov. — Figs 2H, 4N, 5N, 18A–J, 25, 26A, 36A–E

14. *Agroecotettixturneri* sp. nov. — Figs 2K, 4K, 5K, 19A–J, 25, 26A, 36A–D

15. *Agroecotettixquitmanensis* sp. nov. — Figs 2L, 4P, 5P, 20A–J, 25, 26A

16. *Agroecotettixvaquero* sp. nov. — Figs 2T, 4P, 5P, 21A–J, 25

17. *Agroecotettixforcipatus* sp. nov. — Figs 2O, 4O, 5O, 22A–J, 25

18. *Agroecotettixidic* sp. nov. — Figs 2S, 4Q, 5Q, 23A–J, 25, 26B

19. *Agroecotettixkahloae* sp. nov. — Figs 2Q, 4R, 5R, 24A–J, 25, 26B

##### ﻿Species accounts

### ﻿ Agroecotettix modestus

Taxon classificationAnimaliaOrthopteraAcrididae

Bruner, 1908 stat. nov.

830BD8FD-464A-554D-B040-62D228D0B319

Figs 6 , 25

Agroecotettix modestus Bruner, L., 1908. Biologia Centrali-Americana 2: 312.
Agroecotettix modestus modestus Bruner, 1908: Fontana et al. 2008: 155.
Agroecotettix modestus modestus Bruner, 1908: Barrientos-Lozano et al. 2013b: 211–212.

#### Diagnosis.

None of the diagnostic characters used here or typically in the Melanoplinae for species level diagnosis are available as this species is known only from the female type.

#### Female measurements (mm.

(*n* = 1) Body length 28.7; pronotum length 6.9; tegmen length 5.0; hind femur length 15.5; dorsal ovipositor valve length 2.0; ventral ovipositor valve length 2.0.

#### Holotype examined.

• 1♀, Mexico, Durango, Lerdo, November. Deposited in the Academy of Natural Sciences of Drexel University.

#### Habitat.

Bruner (1908) did not report any habitat or environmental data, but it is likely desert scrub as with other members of the genus.

#### Distribution.

Known only from the type locality (Fig. 25).

#### Note.

Given that the only known specimen of this species is female, and it is a distributional outlier with other species occurring between its distribution and that of its subspecies, *A.modestus* is raised to species level.

#### Etymology.

*modestus* Latin = modest.

#### Suggested common name.

Modest aridland scrub jumper.

### ﻿ Agroecotettix silverheelsi sp. nov.

Taxon classificationAnimaliaOrthopteraAcrididae

B71A4D96-4555-543B-871C-9448605694AF

https://zoobank.org/27EE581C-3C87-4296-A82B-536AAC187499

Figs 2B , 4A , 5A , 7A–J , 25 , 23A–C , 25 , 26A

#### Diagnosis.

Differentiated from the other species in the group based on the male cerci that have the ventral branch longer than the dorsal branch and by the male aedeagus that has a thickened sheath, and the valves of that are shorter than the sheath with broadly rounded apices (Figs 4A, 5A). Most similar to *A.aristus* and *A.texmex* but differs from those by the shape of the male genitalia which in caudal view, has the valves of the aedeagus relatively shorter and broader than in *A.aristus* (Figs 4B, 5B) and by the having valves that that are parallel (Figs 4A, 5A) as opposed to diverging apically as in *A.texmex*. (Figs 4D, 5D); in lateral view, the distal edge of the valves is broadly rounded.

#### Male measurements (mm).

(*n* = 7) Body length 20.6–24.5 (mean = 22.7); pronotum length 4.4–5.5 (mean = 5.2); tegmen length 2.9–4.0 (mean = 3.4); hind femur length 10.4–12.4 (mean = 11.6); cerci length 1.4–1.7 (mean = 1.6); basal width of cercus 0.5–0.6 (mean = 0.6); mid-cercal width 0.4 (mean = 0.4); cerci dorsal fork length 0.4–0.5 (mean = 0.5); cerci dorsal fork apex width 0.2 (mean = 0.2); cerci ventral fork length 0.6–0.8 (mean = 0.6); cerci ventral fork apex width 0.1 (mean = 0.1).

#### Phallus measurements (mm).

(*n* = 3) Length 0.7–0.8 (mean = 0.8); apex width 0.4–0.5 (mean = 0.4); middle width 0.5–0.8 (mean = 0.7); basal width 0.6–0.7 (mean = 0.6); lateral apex width 0.2–0.4 (mean = 0.3); lateral medial width 0.3–0.4 (mean = 0.3); lateral basal width 05–0.7 (mean = 0.6).

#### Female measurements (mm).

(*n* = 3) Body length 25.2–27.2 (mean = 26.2); pronotum length 6.5–7.2 (mean = 6.8) tegmen length 4.5–5.1 (mean = 4.8); hind femur length 13.2–15.2 (mean = 14.2); dorsal ovipositor valve length 1.2–2.1 (mean = 1.8); ventral ovipositor valve length 1.2–2.1 (mean = 1.8).

#### Holotype.

• 1♂, USA, Texas, Dimmit Co., Asherton, 28.4559, -99.7778, 19 July 2020, J.G. Hill; open grassland with *Opuntia*, *Cylindropuntia*, and *Prosopis*. Deposited in the Mississippi Entomological Museum.

#### Specimens examined.

USA, **Texas**: • Dimmit Co., Asherton, 28.4559, -99.7778, 19 July 2020, J.G. Hill (1♂, 1♀). • Maverick Co., 1.8 mi E Eagle Pass, 18 August 1961, I.J. Cantrall and T.J. Cohn (5♂, 2♀).

#### Habitat.

On *Vachellia* branches in an open grassland with *Opuntia*, *Cylindropuntia*, and *Prosopis* (Fig. 26C).

#### Distribution.

Known only from the northwestern South Texas Plains region of Texas (Fig. 25).

#### Etymology.

The name *silverheelsi* is a patronym honoring Jay Silverheels. a Native American athlete and actor who most famously portrayed the fictional character Tonto in “The Lone Ranger” television series from 1949–1957. Silverheels was one of the first Native American actors to portray a major Indigenous character on a television series. Throughout his career, Silverheels advocated for more inclusion of Indigenous people in media and founded the Indian Actors Workshop in Los Angeles during the 1960’s. This naming honors Silverheels’ cultural impact and the Texas landscapes where the series was set.

#### Suggested common name.

Silverheels’ aridland scrub jumper.

### ﻿ Agroecotettix aristus

Taxon classificationAnimaliaOrthopteraAcrididae

Hebard, 1922 stat. nov.

F63730B5-60D9-5406-8EB7-E1277D5E75D9

Figs 2A , 4B , 5B , 8A–J , 25 , 26A , 28A–E , 29A–E

Agroecotettix modestus aristus Hebard, 1922. Trans. Amer. Entomol. Soc. 48(1): 49.

#### Diagnosis.

Differentiated from other species in the genus by the combination of male cerci that have the lower branch longer than the dorsal branch and the male aedeagus that has a thickened sheath and valves that are longer than the sheath and have narrowly rounded apices (Figs 4B, 5B). Most similar to *A.silverheelsi* and *A.texmex* but differ from those by the shape of the male genitalia which in caudal view, has the valves of the aedeagus longer and narrower than in *A.silverheelsi* (Figs 4A, 5A) and parallel and narrowly rounded apices as opposed to the broad, latterly diverging apices of *A.texmex* (Figs 4D, 5D). In lateral view, the distal edge of the valves is broadly rounded as in Fig. 5B.

**Figure 8.**
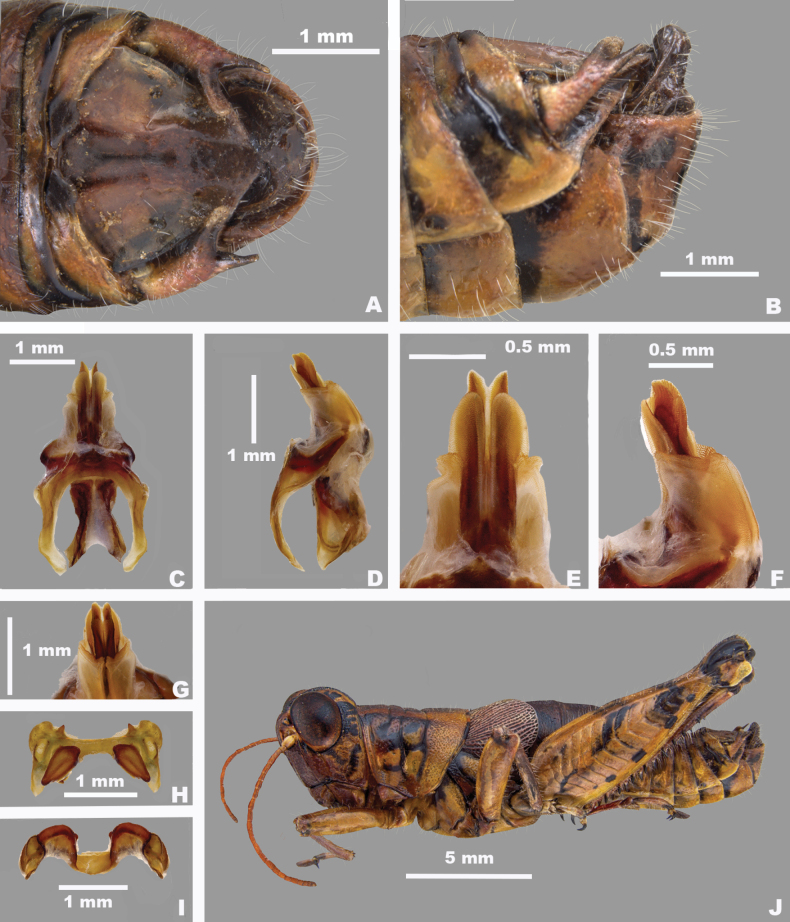
*Agroecotettixaristus***A** dorsal view of male terminalia **B** lateral view of male terminalia **C** dorsal view of phallic complex **D** lateral view of phallic complex **E** dorsal view of aedeagus **F** lateral view of aedeagus **G** caudal view of the aedeagus **H** dorsal view of epiphallus **I** caudal view of epiphallus **J** habitus.

#### Male measurements (mm).

(*n* = 16) Body length 19.5–24.9 (mean = 22.4); pronotum length 4.4–6.2 (mean = 5.2); tegmen length 2.7–4.6 (mean = 3.4); hind femur length 10.4–14.0 (mean = 11.7); cerci length 1.2–1.7 (mean = 1.5); basal width of cercus 0.5–0.7 (mean = 0.6); mid-cercal width 0.4–0.5 (mean = 0.4); cerci dorsal fork length 0.3–0.5 (mean = 0.4); cerci dorsal fork apex width 0.1–0.3 (mean = 0.2) cerci ventral fork length 0.4–0.6 (mean = 0.5); cerci ventral fork apex width 0.1 (mean = 0.1).

#### Phallus measurements (mm).

(*n* = 16) Length 0.6–0.8 (mean = 0.7); apex width 0.3–0.5 (mean = 0.3); middle width 0.4–0.6 (mean = 0.5); basal width 0.5–0.8 (mean = 0.6); lateral apex width 0.2–0.3 (mean = 0.3); lateral medial width 0.3–0.4 (mean = 0.4); lateral basal width 0.5–0.6 (mean = 0.5).

#### Female measurements (mm).

(*n* = 9) Body length 25.5–27.6 (mean = 26.9); pronotum length 6.5–7.3 (mean = 7.0) tegmen length 3.3–6.5 (mean = 4.4); hind femur length 13.3–15.5 (mean = 14.7); Dorsal ovipositor valve length 1.5–2.0 (mean = 1.8); ventral ovipositor valve length 1.5–2.0 (mean = 1.8).

#### Holotype.

• 1♂, USA, Texas, Uvalde, 22 August 1912, Rehn and Hebard, 1000–1100 ft.

#### Specimens examined.

USA, **New Mexico**: • Eddy Co., Sitting Bull Falls, 22 August 1985, B. Ruish, Whiting, (1♂) • Lincoln National Forest, Sitting Bull Falls, 32.2461, -104.6979, 27 September 2024, J.G. Hill (2♂, 1♀). **Texas**: • Culberson Co., Frijole, 4–16 July 1935, J.M. Brennan (1♂) • Jeff Davis Co., Davis Mountains State Park, 30.5992, -103.9075, 16 July 2023, J.G. Hill (1♂, 1♀) • Kimble Co., 5 mi SW Junction, 5 August 1955, T.J. Cohn, 1750 ft (1♂) • Kinney Co., 2 mi S Brackettville, 30 July 1959, T.J. Cohn, 1100 ft (1♂) • Mitchell Co., 1 mi W Colorado City, 9 July 1956, T.J. Cohn, E. Matthews, 2100 ft (2♂) • Odessa Co., Sheffield, 23 October 1931, L. Seaton (1♂, 1♀) • Sterling Co., 7 mi NE Sterling City, 27 June 1967, T.J. Cohn (1♂) • Upton Co., 8.8 mi W. Rankin, 31.1533, -102.0650, 16 July 2023, J.G. Hill, J.L. Seltzer (1♂) • Terrell Co., 18 mi S Sheffield, 1 June 1949, W.F. Blair (1♂, 1♀) • Uvalde Co., Concan, 6 July 1936, R.H. Beamer (1♂) • Pecos Co., 6 mi W Ft. Stockton, 8 August 1956, T.J. Cohn 3000 ft. (2♂) • Uvalde, 22 August 1912, Rehn and Hebard, 1000–1100 ft (Paratypes) (1♂, 1♀) • 21 mi N Uvalde, 29.4636, -100.01389, 29 July 2020, M.J. Thorn, J.G. Hill (1♂, 2♀) • Val Verde Co., 6.5 mi SE Comstock, 23 August 1956, T.J. Cohn, 1400 ft (1♂) • 22 mi NW (rd.) Loma Alta, 31 August 1958 T.J. Cohn (1♂) • Pecos River x HWY 90, 29.705, -101.35084, 24 July 2020, J.G. Hill (1♂, 1♀).

#### Habitat.

Often found on or associated with thorny leguminous shrubs. On the Edwards Plateau in Texas, this species is often found on stunted, low shrubs growing just above ground level amongst the limestone rocks (Fig. 28A–F). In the Davis Mountains and the northern Chihuahuan Desert, this species was observed on the interior branches of larger (> 2 m tall) *Vachellia* species (Fig. 29A–E).

#### Distribution.

Found across central and west Texas to southeastern New Mexico (Figs 25, 26A).

#### Note.

Given that the only known specimen of *A.modestus* is female and it is a distributional outlier, with other species occurring between its distribution and that of its subspecies, *A.modestus* was raised to species level above. Additionally, due to the differences in the internal male genitalia, *A.aristus* and *A.crypsidomus* are each raised to species level.

#### Etymology.

Hebard (1922) did not indicate the etymology in the description of this species, but it is likely from Latin *arista* in reference to the aristate or awn-like point of the male cerci in this genus.

#### Suggested common name.

Aristate aridland scrub jumper.

### ﻿ Agroecotettix xiphophorus sp. nov.

Taxon classificationAnimaliaOrthopteraAcrididae

995A8353-D8F4-54E1-B04C-31E19344C733

https://zoobank.org/D5ABD4CA-455E-4C03-8C3A-F6BF29A1147C

**Figure 9.**
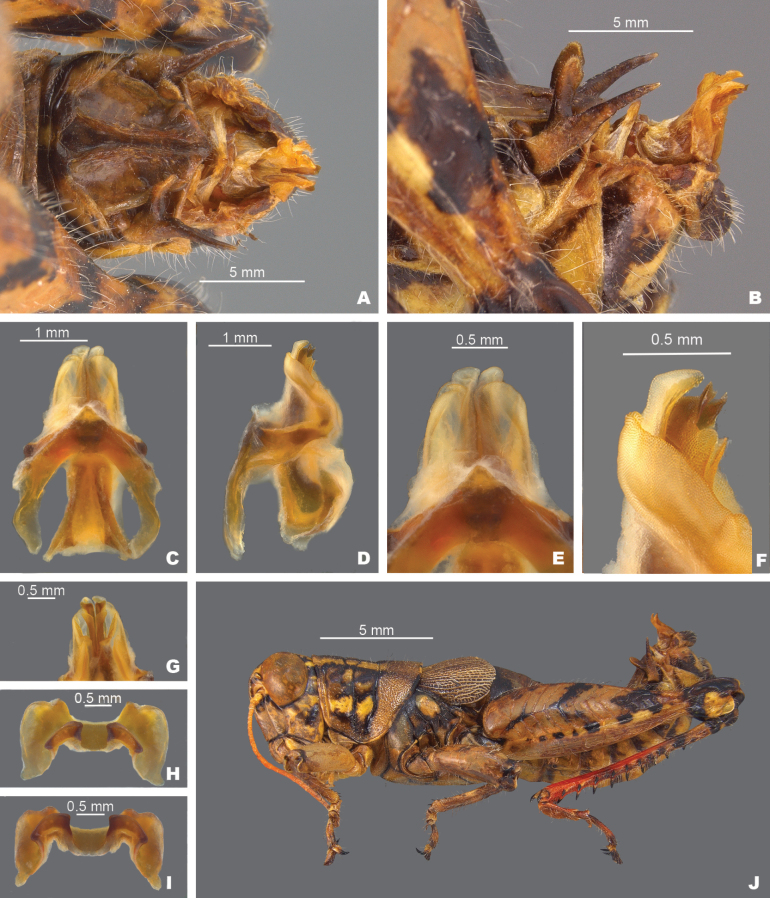
*Agroecotettixxiphophorus***A** dorsal view of male terminalia **B** lateral view of male terminalia **C** dorsal view of phallic complex **D** lateral view of phallic complex **E** dorsal view of aedeagus **F** lateral view of aedeagus **G** caudal view of the aedeagus **H** dorsal view of epiphallus **I** caudal view of epiphallus **J** habitus.

Figs 2C, D , 4C , 5C , 9A–J , 25 , 26B

#### Diagnosis.

Easily differentiated from other species in the genus by the combination of male cerci with branches widely separated and with the ventral branch much longer than the dorsal branch (Figs 2C, D, 9A, B), the male aedeagus that has a thickened sheath and valves with somewhat bilobed dorsal margins and broadly rounded ventral margins that have with their distal apices diverging laterally (Figs 4C, 5C). Most like *A.idic* and *A.kahloae* but is distinguished from those species by the shape of the male cerci, which in *A.idic* and *A.kahloae* are smaller with the dorsal and ventral branches of similar length, and by the shape of the male phallic complex (Figs 9C–G, 23C–G, 24C–G).

#### Male measurements (mm).

(*n* = 8) Body length 21.8–27.5 (mean = 24.8); pronotum length 5.0–6.7 (mean = 5.9); tegmen length 3.5–4.6 (mean = 4.1); hind femur length 11.7–14.1 (mean = 12.9); cerci length 2.0–2.5 (mean = 2.3); basal width of cercus 0.5–0.7 (mean = 0.6); mid-cercal width 0.3–0.7 (mean = 0.6); cerci ventral arm length 1.2–1.7 (mean = 1.5); cerci ventral arm apex width 0.1 (mean = 0.1) cerci dorsal arm length 0.6–0.9 (mean = 0.8); cerci dorsal arm apex width 0.2–0.4 (mean = 0.3).

#### Phallus measurements (mm).

(*n* = 5) Length 0.7–1.0 (mean = 0.8); apex width 0.4–0.5 (mean = 0.5); middle width 0.9–1.0 (mean = 0.9); basal width 0.6–0.7 (mean = 0.7); lateral apex width 0.2–0.4 (mean = 0.3); lateral medial width 0.4–0.6 (mean = 0.5); lateral basal width 0.5–0.9 (mean = 0.7).

#### Female measurements (mm).

(*n* = 7) Body length 24.0–31.3 (mean = 27.5); pronotum length 6.7–8.8 (mean = 7.5); tegmen length 4.1–5.3 (mean = 4.7); hind femur length 14.4–16.7 (mean = 15.5) Dorsal ovipositor valve length 1.6–2.5 (mean = 2.0); ventral ovipositor valve length 1.6–2.5 (mean = 2.0).

#### Holotype.

• 1♂, Mexico, Nuevo Leòn, 1.7 mi W Santa Caterina. 8 August 1959, T.J. Cohn, 2380 ft, #155. Deposited in the Mississippi Entomological Museum.

#### Specimens examined.

Mexico, **Nuevo Leòn**: • Monterrey airport, 14 July 1964, T.J. Cohn (2♂, 1♀) • 5 mi W Monterrey, 16 July 1936, H.R. Roberts, 3000 ft. (2♂, 2♀) • Villa de Garcia, 28–29 August 1966, J. Mathieu (4♂, 3♀).

#### Habitat.

Cohn (1959) lists the habitat at the type locality as a fair patch of roadside weeds, especially a tall sticky composite and blue flowered solanaceous plant with spaced out low trees of Acacia and mesquite.

#### Distribution.

Found west-central Nuevo Leòn, Mexico (Figs 25, 26B).

#### Etymology.

*xiphos* Greek = sword; *phorus* Greek = bearing:. reference to the long sword-like ventral projection of the male cerci.

#### Suggested common name.

Sword-tailed aridland scrub jumper.

### ﻿ Agroecotettix glochinos sp. nov.

Taxon classificationAnimaliaOrthopteraAcrididae

C6395B04-11D7-5E05-84C1-82509C325BCA

https://zoobank.org/AEE452D0-59FA-4F1F-B50B-51EA3630666B

**Figure 10.**
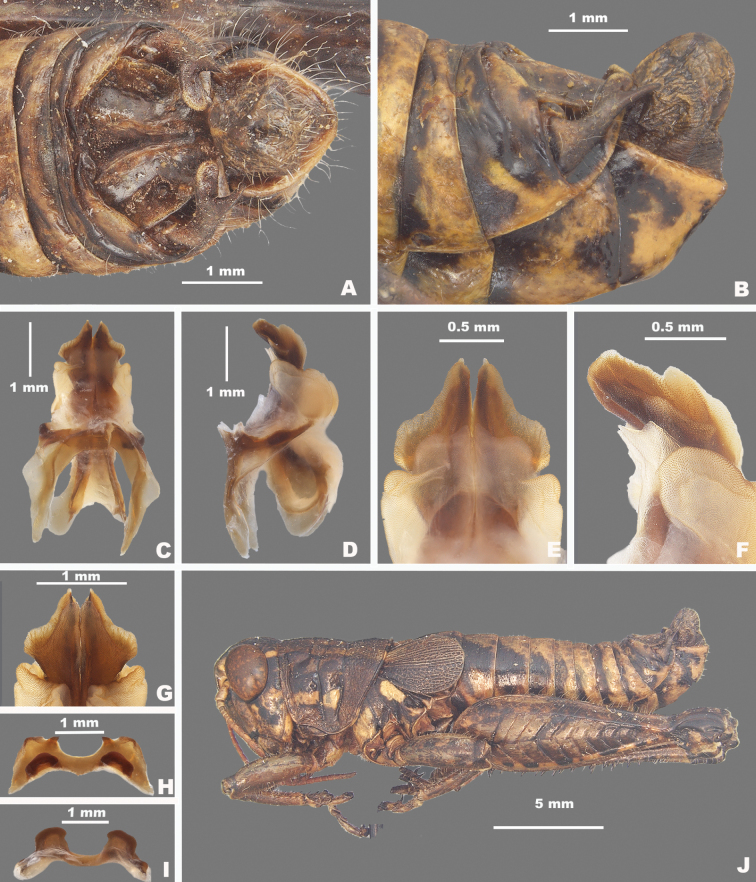
*Agroecotettixglochinos***A** dorsal view of male terminalia **B** lateral view of male terminalia **C** dorsal view of phallic complex **D** lateral view of phallic complex **E** dorsal view of aedeagus **F** lateral view of aedeagus **G** caudal view of the aedeagus **H** dorsal view of epiphallus **I** caudal view of epiphallus **J** habitus.

Figs 4D , 5D , 10A–J , 25 , 26B

#### Diagnosis.

Easily differentiated from other species in the genus by the combination of male cerci that have the lower branch longer than the dorsal branch (Fig. 10A, B) and the male aedeagus with a thickened sheath, valves that are longer than the sheath, and in caudal view the valves of the aedeagus are widen laterally in their mid-section, and abruptly narrow apically such that the dorsal apieces form acute parallel points; the ventral apices of the valves are broadly rounded (Figs 4D, 10C–G). In lateral view the valves are directed apically instead of dorsally as in all other *Agroecotettix* species (Figs 5D, 10C–G).

#### Male measurements (mm).

(*n* = 2) Body length 13.3–18.7 (mean = 16); pronotum length 2.9–4.1 (mean = 3.5); tegmen length 2.2–3.0 (mean = 2.6); hind femur length 7.3–10.0 (mean = 8.7); cerci length 0.8–1.1 (mean = 1.0); basal width of cercus 0.4–0.5 (mean = 0.5); mid-cercal width 0.2–0.3 (mean = 0.3); cerci dorsal fork length 0.1–0.3 (mean = 0.2); cerci dorsal fork apex width 0.1–0.2 (mean = 0.2); cerci ventral fork length 0.2–0.4 (mean = 0.3); cerci ventral fork apex width 0.1 (mean = 0.1).

#### Phallus measurements (mm).

(*n* = 1) Length 0.8; apex width 0.3; middle width 0.4; basal width 0.6; lateral apex width 0.5; lateral medial width 0.7; lateral basal width 0.7.

#### Female measurements (mm).

(*n* = 3) Body length 21.3–23.3 (mean = 22.0); pronotum length 4.7–5.1 (mean = 4.9) tegmen length 3.5–3.8 (mean = 3.7); hind femur length 10.8–11.7 (mean = 11.2); dorsal ovipositor valve length 1.3–1.5 (mean = 1.3); ventral ovipositor valve length 1.3–1.5 (mean = 1.4).

#### Holotype.

• 1♂, Mexico, Nuevo Leòn, 7 mi SE Galeana, 11 August 1959, 5350’, T.J. Cohn, #166. Deposited in the Mississippi Entomological Museum.

#### Specimens examined.

Mexico, **Nuevo Leòn**: • 7 mi SE Galeana, 11 August 1959, T.J. Cohn, 5350’ (1♂, 3♀).

#### Habitat.

Cohn (1959) does not include a habitat description for the single locality from which this species is known.

#### Distribution.

Known only from the type locality at this time (Figs 25, 26B).

#### Etymology.

*glochinos* Greek = point of an arrow, in reference to the pointed, arrowhead-like shape of the aedeagus.

#### Suggested common name.

Arrowhead aridland scrub jumper.

### ﻿ Agroecotettix texmex sp. nov.

Taxon classificationAnimaliaOrthopteraAcrididae

325DC5FD-38CF-55B8-A4B2-633F89665470

https://zoobank.org/04E23B25-B968-450D-9988-E6D7FE14622E

**Figure 11.**
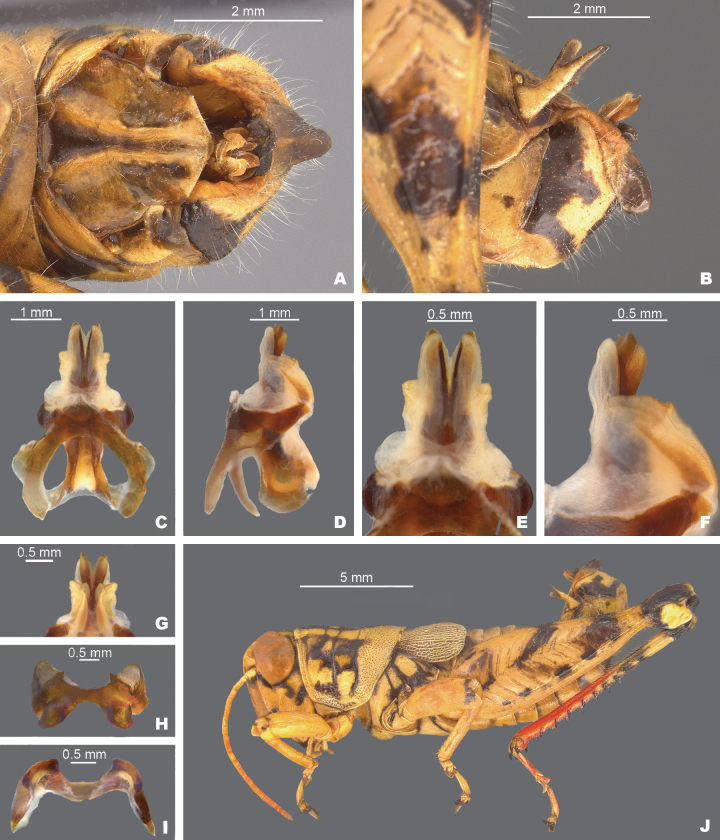
*Agroecotettixtexmex***A** dorsal view of male terminalia **B** lateral view of male terminalia **C** dorsal view of phallic complex **D** lateral view of phallic complex **E** dorsal view of aedeagus **F** lateral view of aedeagus **G** caudal view of the aedeagus **H** dorsal view of epiphallus **I** caudal view of epiphallus **J** habitus.

Figs 2E , 4E , 5E , 11A–J , 25 , 26B

#### Diagnosis.

Differentiated from other species in the genus by the combination of male cerci that have the lower branch longer than the dorsal branch (Figs 2E, 11A, B) and the male aedeagus that has a thickened sheath and valves that are longer than the sheath, and narrow valves that diverge in lateral view (Figs 4E, 5E, 11C–G). Most similar to *A.aristus*, *A.glochinos*, and *A.silverheelsi*. Differs from *A.aristus* by that species by having valves that diverge laterally in caudal view and are broader in lateral view (Figs 4C, D, 5C, D) and from *A.glochinos* by having narrower valves (Figs 4D, E, 5D, E). Differs from *A.silverheelsi* by having a narrower and folded caudal edge of the sheath as opposed to an unfolded and open edge as in *A.silverheelsi*.

#### Male measurements (mm).

(*n* = 7) Body length 22.2–24.0 (mean = 23.1); pronotum length 4.6–6.5 (mean = 5.4); tegmen length 3.3–4.1 (mean = 3.6); hind femur length 11.5–13.5 (mean = 12.6); cerci length 1.6–1.8 (mean = 1.7); basal width of cerci 0.4–0.7 (mean = 0.6); mid-cercal width 0.4–0.6 (mean = 0.5); cerci dorsal fork length 0.4–0.7 (mean = 0.5); cerci dorsal fork apex width 0.1–0.3 (mean = 0.2); cerci ventral fork length 0.4–1.0 (mean = 0.7); cerci ventral fork apex width 0.1 (mean = 0.1).

#### Phallus measurements (mm).

(*n* = 2) Length 0.6–0.7 (mean = 0.7); apex width 0.4–0.5 (mean = 0.5); middle width 0.5–0.6 (mean = 0.6); basal width 0.3 (mean = 0.3); lateral apex width 0.2–0.4 (mean = 0.3); lateral medial width 0.2–0.5 (mean = 0.4); lateral basal width 0.2–0.6 (mean = 0.4).

#### Female measurements (mm).

(*n* = 7) Body length 25.2–29.8 (mean = 27.3); pronotum length 6.5–7.5 (mean = 6.9) tegmen length 4.1–5.1 (mean = 4.7); hind femur length 14.0–16.2 (mean = 15.1); dorsal ovipositor valve length 1.5–2.0 (mean = 1.7); ventral ovipositor valve length 0.6–2.0 (mean = 1.6).

#### Holotype.

• 1♂, USA, Texas, Jim Hogg Co., 26 mi S Hebronville, 19 August 1955, 5–700 ft., T.J. Cohn. Deposited in the Mississippi Entomological Museum.

#### Specimens examined.

Mexico, **Tamaulipas**: • 12 mi S Nuevo Laredo, 9 July 1936, H.R. Roberts (1♂, 1♀) • Nuevo Leòn, 34 miles S Sabinas Hildalgo, 12 IX 1958, 1700 ft, T.J. Cohn (1♂, 1♀) • Mamulique Pass, 10 July 1936, 1800 ft, H.R. Roberts (2♀); 19 mi W Santa Catarina, 9 August 1959, T.J. Cohn (♂) • 6 mi SE Santiago, 29 September 1958.T.J. Cohn, 1550 ft (1♂, 1♀) • Villa de Santiago, 4 July 1964, T.J. Cohn (1♂).

#### Habitat.

Cohn (1959) states the habitat at 19 mi W of Santa Catarina was above an arroyo in an area with large smooth margined leaved oaks and a variety of low bushes, succulents, broadleaf blackberry, and sparse but good weeds in clumps. The habitat at Villa de Santiago was rolling country in spined bushes that were fairly thick and more than 8 feet tall (Cohn 1964).

#### Distribution.

Southern Texas and northeastern Mexico (Figs 25, 26B).

#### Etymology.

The name *texmex* is a portmanteau of Texas and Mexico as this is the only known species of *Agroecotettix* that occurs in both countries.

#### Suggested common name.

Texmex aridland scrub jumper.

### ﻿ Agroecotettix cumbres sp. nov.

Taxon classificationAnimaliaOrthopteraAcrididae

1A14675F-A6DD-5B94-8E33-24095F636F8C

https://zoobank.org/FEE97243-6929-491A-9BC4-7020291EAFD4

**Figure 12.**
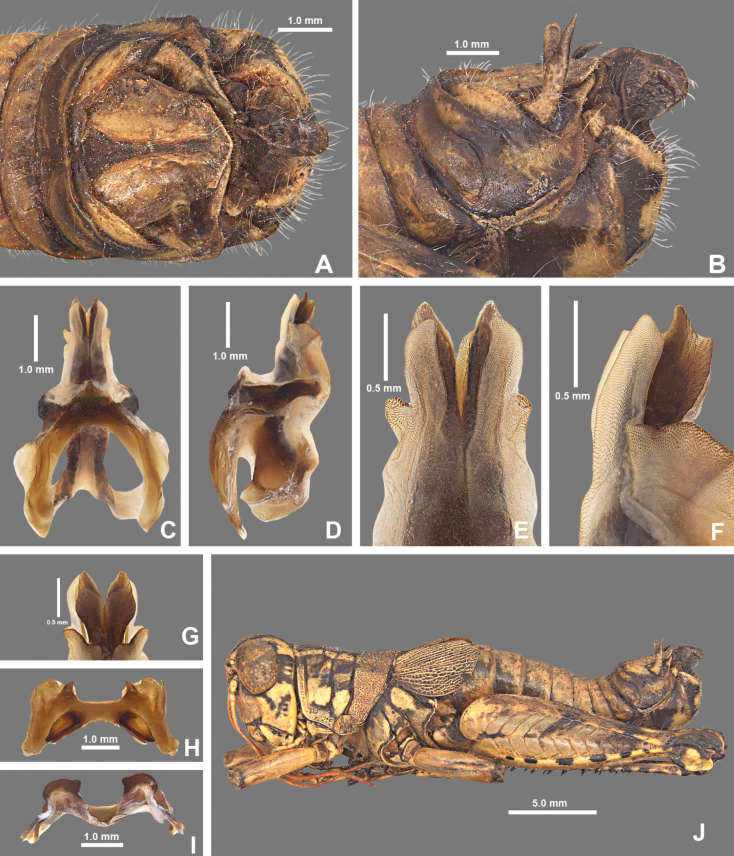
*Agroecotettixcumbres***A** dorsal view of male terminalia **B** lateral view of male terminalia **C** dorsal view of phallic complex **D** lateral view of phallic complex **E** dorsal view of aedeagus **F** lateral view of aedeagus **G** caudal view of the aedeagus **H** dorsal view of epiphallus **I** caudal view of epiphallus **J** habitus.

Figs 2F , 4F , 5F , 12A–J , 25 , 26B

#### Diagnosis.

Differentiated from other species in the genus by the combination of male cerci that have the lower branch longer than the dorsal branch (Figs 2F, 12A, B) and the male aedeagus that has a thickened sheath, valves that are longer than the sheath, and broad valves that have their distal half angled caudally (Figs 4F, 5F, 12C–G). Most similar to *A.texmex* but differs by having valves that are broader than that species both in lateral and caudal view (Figs 4D, E, 5D, E).

#### Male measurements (mm).

(*n* = 4) Body length 21.3–23.5 (mean = 22.5); pronotum length 4.7–5.5 (mean = 5.0); tegmen length 3.2–4.2 (mean = 3.7); hind femur length 11.1–12.2 (mean = 11.8); cerci length 1.7–1.9 (mean = 1.8); basal width of cercus 0.5–0.6 (mean = 0.6); mid-cercal width 0.4–0.5 (mean = 0.5); cerci dorsal fork length 0.5–0.7 (mean = 0.6); cerci dorsal fork apex width 0.5–0.7 (mean = 0.6); cerci ventral fork length 0.7–0.9 (mean = 0.8); cerci ventral fork apex width 1.0 (mean = 1.0).

#### Phallus measurements (mm).

(*n* = 4) Length 0.6–0.8 (mean = 0.8); apex width 0.3–0.4 (mean = 0.4); middle width 0.5–0.6 (mean = 0.6); basal width 0.5 (mean = 0.5); lateral apex width 0.2–0.3 (mean = 0.3); lateral medial width 0.4–0.5 (mean = 0.5); lateral basal width 0.6 (mean = 0.6).

#### Female measurements (mm).

(*n* = 11) Body length 24.1–28.0 (mean = 25.7); pronotum length 5.5–7.0 (mean = 6.4) tegmen length 4.0–5.0 (mean = 4.4); hind femur length 12.1–15.5 (mean = 14.3); dorsal ovipositor valve length 1.4–2.0 (mean = 1.7); ventral ovipositor valve length 1.4–2.0 (mean = 1.7).

#### Holotype.

• 1♂, Mexico, Nuevo Leòn, 500–800 m. 24 mi NW Montemorelos, 3 Sept. 1955, T.J. Cohn. Deposited in the Mississippi Entomological Museum.

#### Specimens examined.

Mexico, **Nuevo Leòn**: • 10 mi NW Montemorelos, 29 September 1958, T.J. Cohn, 2000 ft (1♂, 1♀) • 24 mi NW Montemorelos, 3 September 1955, T.J. Cohn (11♂, 2♀) • 5 mi SW Santiago, Horse Tail Falls, 29 September 1958. T.J. Cohn (1♂) • 6 mi SW Villa Santiago, 29 September 1958. T.J. Cohn (1♂, 3♀).

#### Distribution.

Area to the south of Monterrey, Mexico and in and east of Monterrey Peaks (Figs 25, 26B).

#### Habitat.

Cohn (1955) describes the locality at 24 mi NW Montemorelos as a rocky hillside with a draw and thorny bushes and at 6 mi SW Villa de Santiago as rolling as country in heavy spiney bushes, fairly thick and more than 8 ft tall. Cohn (1964) describes the locality at the Monterrey airport as badly overgrazed range, but with a good variety of green, thick, bushes, including fair-sized mesquite and other leguminous trees, soil sloped.

#### Etymology.

The specific epithet *cumbres* is the Spanish word for summits and is in reference to the Parque Nacional Cumbres de Monterrey and the mountain summits near where this species found.

#### Suggested common name.

Cumbres aridland scrub jumper.

### ﻿ Agroecotettix crypsidomus

Taxon classificationAnimaliaOrthopteraAcrididae

Hebard, 1922 stat. nov.

F88C0D25-7D72-50A7-9234-E7DFCEF3510C

**Figure 13.**
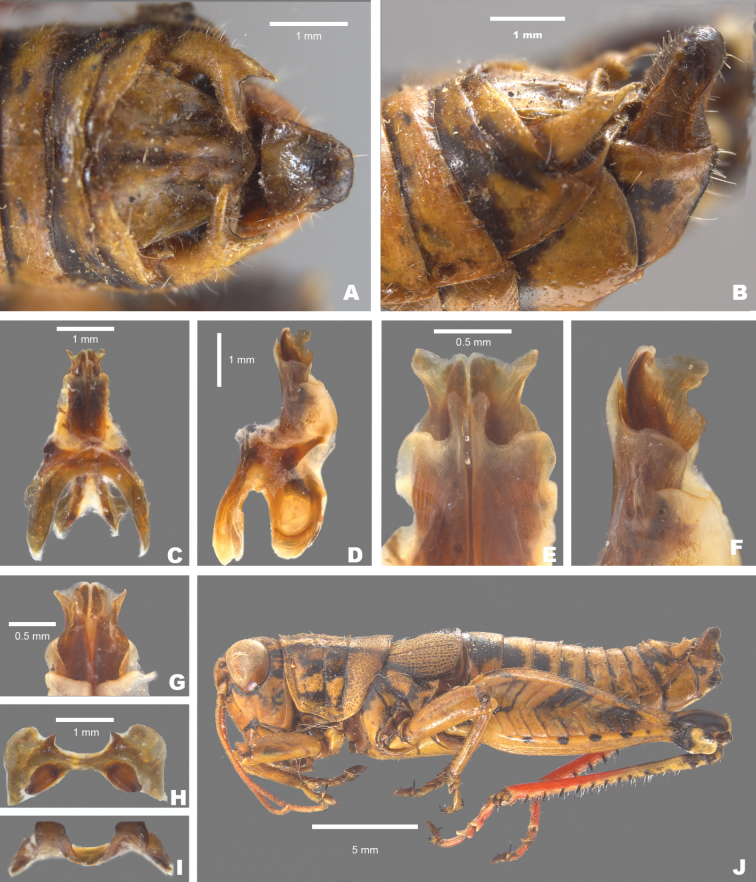
*Agroecotettixcrypsidomus***A** dorsal view of male terminalia **B** lateral view of male terminalia **C** dorsal view of phallic complex **D** lateral view of phallic complex **E** dorsal view of aedeagus **F** lateral view of aedeagus **G** caudal view of the aedeagus **H** dorsal view of epiphallus **I** caudal view of epiphallus **J** habitus.

Figs 2G , 4G , 5G , 13A–J , 25 , 26A , 31A–F

Agroecotettix modestus crypsidomus Hebard, 1922. Trans. Amer. Entomol. Soc. 48(1): 53.

#### Diagnosis.

Differentiated from other species in the genus by the combination of male cerci with ventral branch equal or subequal in length to dorsal branch as in Fig. 2G; thin and lightly sclerotized sheath (Fig. 5H); valves of the aedeagus that are lobate with the basal lobe more produced in lateral view, extending beyond the sheath as in Fig. 4G, and with the dorsal lobe projected laterally in caudal view as in Fig. 5G.

#### Male measurements (mm).

(*n* = 11) Body length 18.0–21.5 (mean = 23.8); pronotum length 4.2–5.5 (mean = 4.7); tegmen length 2.8–3.9 (mean = 3.3); hind femur length 9.6–11.7 (mean = 11.0); cerci length 1.2–1.5 (mean = 1.4); basal width of cercus 0.5–0.7 (mean = 0.6); mid-cercal width 0.3–0.5 (mean = 0.4); cerci dorsal fork length 0.4–0.6 (mean = 0.5); cerci dorsal fork apex width 0.2 (mean = 0.2) cerci ventral fork length 0.3–0.5 (mean = 0.4); cerci ventral fork apex width 0.1 (mean = 0.1).

#### Phallus measurements (mm).

(*n* = 11) Length 1.0–1.1 (mean = 1.1); apex width 0.3–0.7 (mean = 0.5); middle width 0.4–0.5 (mean = 0.4); Basal width 0.6–0.7 (mean = 0.7); lateral apex width 0.4–0.6 (mean = 0.6); lateral medial width 0.5–0.6 (mean = 0.5); lateral basal width 0.5–0.7 (mean = 0.6).

#### Female measurements (mm).

(*n* = 14) Body length 21.0–25.7 (mean = 23.8); pronotum length 4.9–6.7 (mean = 5.8) tegmen length 3.1–4.5 (mean = 3.9); hind femur length 11.3–14.8 (mean = 13.2); Dorsal ovipositor valve length 0.9–2.0 (mean = 1.6); ventral ovipositor valve length 0.9–2.0 (mean = 1.6).

#### Holotype examined.

• 1♂, USA, Texas, Marathon, Brewster Co., Sept. 12–13, 1912, Rehn and Hebard, 2000–4160 ft. Deposited in the Academy of Natural Sciences of Drexel University.

#### Specimens examined.

USA, **Texas**: • Garden Springs, 2 September 1912, Rehn and Hebard (1♂, 1♀) • 4 mi S Marathon, 11 October 1952, M.J.D. White (3♀) • 4.3 mi S Marathon, 30.1530, -103.2865, 13 July 2023, J.G. Hill, J.L. Seltzer (2♂, 2♀) • Marathon, 12–13 September 1912, Rehn and Hebard (9♂, 9♀).

#### Habitat.

Chihuahuan Desert scrub, often associated with thorny shrubs such as *Acacia* (Fig. 31A–F).

#### Distribution.

Found in the vicinity of Marathon, Texas and the Marathon basin (Figs 25, 26A).

#### Note.

Given that the only known specimen of *A.modestus* is female and it is a distributional outlier, with other species occurring between its distribution and that of its subspecies, *A.modestus* was raised to species level above. Additionally, due to the differences in the internal male genitalia, *A.aristus* and *A.crypsidomus* are each raised to species level.

#### Etymology.

*crypsi* Greek = hidden, *domus* Latin = home: in reference to the cryptic nature of the species living in the inner branches of thorny shrubs.

#### Suggested common name.

Seclusive aridland scrub jumper.

### ﻿ Agroecotettix burtoni sp. nov.

Taxon classificationAnimaliaOrthopteraAcrididae

1D997586-2134-54CF-B794-FFCEA33A68F4

https://zoobank.org/3021D508-7E34-41ED-8AE8-1E18BC603211

**Figure 14.**
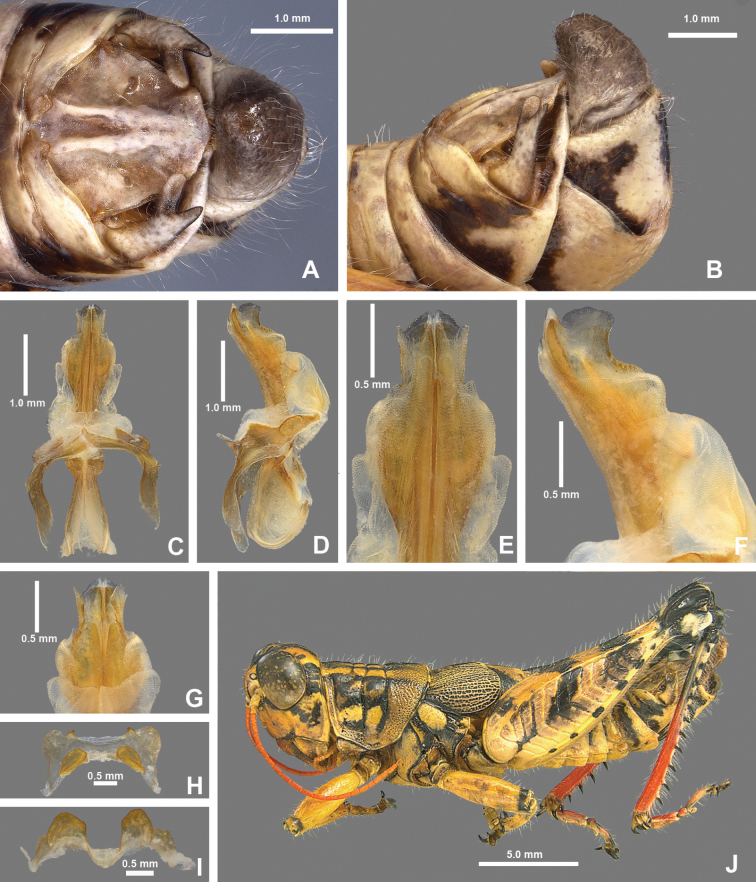
*Agroecotettixburtoni***A** dorsal view of male terminalia **B** lateral view of male terminalia **C** dorsal view of phallic complex **D** lateral view of phallic complex **E** dorsal view of aedeagus **F** lateral view of aedeagus **G** caudal view of the aedeagus **H** dorsal view of epiphallus **I** caudal view of epiphallus **J** habitus.

Figs 2J , 4H , 5H , 14A–J , 25 , 26A , 31A–D

#### Diagnosis.

Differentiated from other species in the genus by the combination of male cerci with ventral branch equal or subequal in length to dorsal branch (Figs 2J, 14A, B), a thin and lightly sclerotized sheath of aedeagus (Figs 5H, 14C–G); valves of the aedeagus lobate in lateral view with the basal lobe not produced much beyond the sheath in lateral view (Figs 5H, 14D, F) and with the valves not projected laterally in caudal view, being almost vertical or curving medially (Fig. 5H, 14G).

#### Male measurements (mm).

(*n* = 10) Body length 19.5–24.0 (mean = 21.6); pronotum length 4.0–5.0 (mean = 4.6); tegmen length 3.0–4.0 (mean = 3.5); hind femur length 10.5–12.4 (mean = 11.3); cerci length 1.0–1.3 (mean = 1.2); basal width of cercus 0.5–0.6 (mean = 0.6); mid-cercal width 0.3–0.5 (mean = 0.4); cerci ventral projection length 0.3–0.4 (mean = 0.3); cerci ventral projection apex width 0.1 (mean = 0.1) cerci dorsal projection length 0.3–0.4 (mean = 0.3); cerci dorsal projection apex width 0.2–0.3 (mean = 0.2).

#### Phallus measurements (mm).

(*n* = 3) Length 1.1 (mean = 1.1); apex width 0.5 (mean = 0.5); middle width 0.5 (mean = 0.5); Basal width 0.7 (mean = 0.7); lateral apex width 0.5–0.6 (mean = 0.6); lateral medial width 0.4–0.5 (mean = 0.4); lateral basal width 0.4–0.5 (mean = 0.5).

#### Female measurements (mm).

(*n* = 6) Body length 24.0–29.9 (mean = 25.9); pronotum length 5.5–6.5 (mean = 5.8); tegmen length 3.5–4.5 (mean = 4.1); hind femur length 13.0–14.5 (mean = 13.9) dorsal ovipositor valve length 1.5–2.0 (mean = 1.7); ventral ovipositor valve length 1.5–2.0 (mean = 1.7).

#### Holotype.

• 1♂, USA, Texas, Brewster Co., Big Bend N.P., 29.3178, -103.3942, 15 July 2023, J.G. Hill; Collected in Chihuahuan Desert scrub. Deposited in the Mississippi Entomological Museum.

#### Specimens examined.

USA, **Texas**: • Brewster Co., Big Bend N.P., 29.3988, -103.2029, 14 July 2023, J.G. Hill, R.C. Seltzer-Hill (1♂) • Big Bend N.P., 29.3178, -103.3942, 15 July 2023, J.G. Hill (2♀) • 1.4 mi NE Government Springs Junction Big Ben Park, 12 June 1961, T.J. and J.W. Cohn (2♂) • Basin, 8 September 1951, T.J. Cohn (1♂) • Glenn Spring, 1 August 1928, F.M. Gaige (6♂, 3♀).

#### Habitat.

Chihuahuan Desert Scrub (Fig. 31A).

#### Distribution.

Endemic to the Chihuahuan desert and more specifically to the southern big bend region of Texas (Figs 25, 26A). At present, all known populations occur within Big Bend National Park.

#### Etymology.

The species name *burtoni* is a patronym honoring LeVar Burton, an iconic American actor, director, and children’s television host renowned for his influential work in promoting literacy and education, particularly through his long-running role as the host of “Reading Rainbow.” Additionally, Burton is celebrated for his inspirational portrayal of Lieutenant Commander Geordi La Forge in “Star Trek: The Next Generation” and its spin-offs. His contributions to education and his advocacy for intellectual and cultural enrichment make him a fitting namesake for a species that thrives in the Big Bend region of Texas where deep history, nature, and vast starry skies come together in a unique American landscape.

#### Suggested common name.

Burton’s aridland scrub jumper.

### ﻿ Agroecotettix moorei sp. nov.

Taxon classificationAnimaliaOrthopteraAcrididae

4FFCE7BB-06C5-5BC3-8B32-40CCC80FCE8F

https://zoobank.org/9A4847D5-CC4C-4F38-98FA-34CDB373A25C

**Figure 15.**
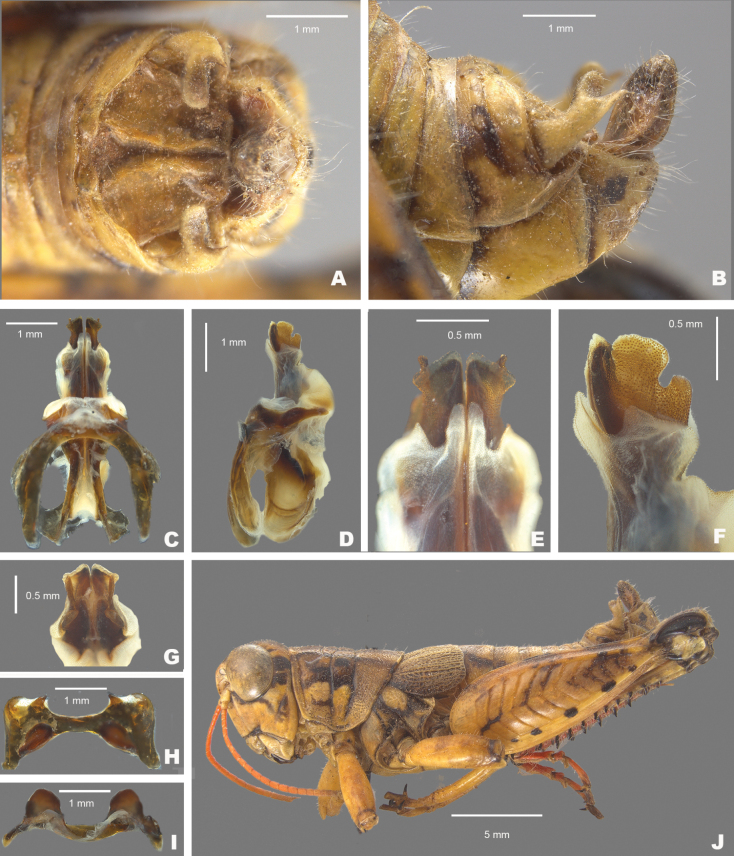
*Agroecotettixmoorei***A** dorsal view of male terminalia **B** lateral view of male terminalia **C** dorsal view of phallic complex **D** lateral view of phallic complex **E** dorsal view of aedeagus **F** lateral view of aedeagus **G** caudal view of the aedeagus **H** dorsal view of epiphallus **I** caudal view of epiphallus **J** habitus.

Figs 2M , 4I , 5I , 15A–J , 25 , 26A , 32A–D

#### Diagnosis.

Differentiated from other species in the genus by the combination of male cerci with ventral branch equal or subequal in length to dorsal branch (Figs 2L, 15A, B); sheath of aedeagus thin and lightly sclerotized (Fig. 5I); valves of the aedeagus are lobate and in lateral view, are shallowly incised with a broad distal lobe that is truncated apically, and the basal lobe is shorter (Figs 5I, 15D, F); in caudal view the valves of the aedeagus are concave as in Fig. 4I. Most like *A.crypsidomus* but differs in shape of the dorsal valves of the male aedeagus which with the broad distal lobe being distinctive for *A.moorei*. Furthermore, when viewed from above the inner margins of the valves of *A.moorei* form a right angle as opposed to being broadly rounded in *A.crypsidomus*.

#### Male measurements (mm).

(*n* = 8) Body length 18.5–24.0 (mean = 20.2); pronotum length 4.3–5.0 (mean = 4.6); tegmen length 2.6–3.5 (mean = 3.0); hind femur length 10.0–12.1 (mean = 10.8); cerci length 1.2–1.3 (mean = 1.3); basal width of cercus 0.5 (mean = 0.5); mid-cercal width 0.3–0.5 (mean = 0.4); cerci dorsal fork length 0.3–0.5 (mean = 0.4); cerci dorsal fork apex width 0.2–0.3 (mean = 0.6) cerci ventral fork length 0.3–0.5 (mean = 0.4); cerci ventral fork apex width 0.1 (mean = 0.1).

#### Phallus measurements (mm).

(*n* = 5) Length 0.9–1.0 (mean = 0.9); apex width 0.5–0.6 (mean = 0.5); middle width 0.4–0.5 (mean = 0.5); Basal width 0.7 (mean = 0.7); lateral apex width 0.4–0.6 (mean = 0.5); lateral medial width 0.5–0.8 (mean = 0.6); lateral basal width 0.6–0.7 (mean = 0.6).

#### Female measurements (mm).

(*n* = 4) Body length 16.2–27.0 (mean = 22.1); pronotum length 4.8–6.5 (mean = 5.6) tegmen length 3.0–4.3 (mean = 3.6); hind femur length 10.8–14.2 (mean = 12.5); Dorsal ovipositor valve length 1.2–1.4 (mean = 1.3); ventral ovipositor valve length 1.2–1.4 (mean = 1.3).

#### Holotype.

• 1♂, USA, Texas, Terrel Co., Sanderson, 30.1485, -102.3977, 30 Jul 2021, J.G. Hill, Collected in Chihuahuan desert. Deposited in the Mississippi Entomological Museum.

#### Specimens examined.

USA, **Texas**: • Terrell Co. Sanderson, 25 August 1912, Rehn and Hebard, 2750–3180’ (4♂, 4♀) • Sanderson, 30.1458, -102.3977, 30 July 2021, Z.D. Brown, (1♂, 1♀).

#### Habitat.

Chihuahuan desert scrub (Fig. 32D).

#### Distribution.

Apparently, a narrow range endemic species that is restricted to the area around Sanderson, Texas in the Chihuahuan Desert (Figs 25, 26A).

#### Etymology.

The species name *moorei* is a patronym honoring Clayton Moore, the American actor who most famously starred as a fictional Texas Ranger in “The Lone Ranger” television series from 1949–1957. Moore’s portrayal of the character embodied qualities of justice, bravery, and a deep connection to the American West. This naming honors Moore’s cultural impact and the desert landscapes that inspired Moore’s legendary character.

#### Suggested common name.

Moore’s aridland scrub jumper.

### ﻿ Agroecotettix chiantiensis sp. nov.

Taxon classificationAnimaliaOrthopteraAcrididae

E59FF2D8-3905-58CA-A85F-136B62ED1B51

https://zoobank.org/0F102A87-0418-4C34-BB5B-EBF28D8CC9DE

**Figure 16.**
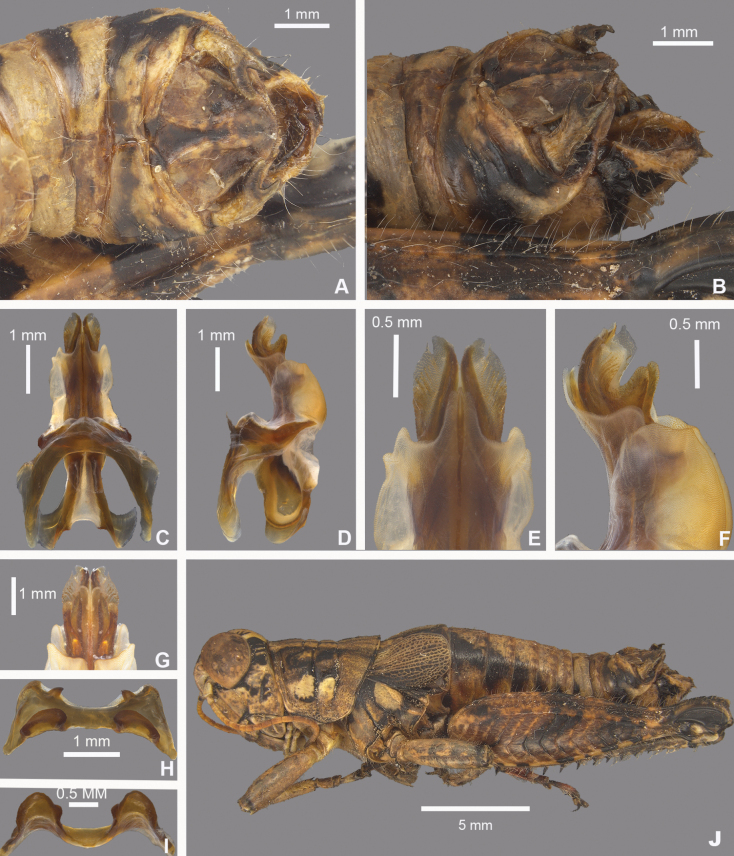
*Agroecotettixchiantiensis***A** dorsal view of male terminalia **B** lateral view of male terminalia **C** dorsal view of phallic complex **D** lateral view of phallic complex **E** dorsal view of aedeagus **F** lateral view of aedeagus **G** caudal view of the aedeagus **H** dorsal view of epiphallus **I** caudal view of epiphallus **J** habitus.

Figs 2N , 4J , 5J , 16A–J , 25 , 26A , 33A–C

#### Diagnosis.

Differentiated from other species in the genus by the combination of male cerci with ventral branch equal or subequal in length to dorsal branch (Figs 2N, 15A, B); sheath of aedeagus thin and lightly sclerotized (Fig. 5J); valves of the aedeagus are lobate, and in lateral view, are deeply incised with a narrower and slightly acute distal lobe and a longer basal lobe as in Figs 5J, 16D, F); in caudal view the valves of the aedeagus are convex as in Figs 4J, 15G).

#### Male measurements (mm).

(*n* = 1) Body length 20.5; pronotum length 4.2; tegmen length 3.2; hind femur length 11.2; cerci length 1.1; basal width of cercus 0.5; mid-cercal width 0.4; cerci dorsal fork length 0.3; cerci dorsal fork apex width 0.2; cerci ventral fork length 0.2; cerci ventral fork apex width 1.

#### Phallus measurements (mm).

(*n* = 1) Length 1; apex width 0.4; middle width 0.4; basal width 0.6; lateral apex width 0.5; lateral medial width 0.7; lateral basal width 0.7.

#### Female measurements (mm).

(*n* = 1) Body length 25.1; pronotum length 6.2; tegmen length 3.7; hind femur length 15.1; dorsal ovipositor valve length 2; ventral ovipositor valve length 2.

#### Holotype.

• 1♂, USA, Texas, Presidio Co., 32 mi SW Marfa, 30.0488, -104.4663, 15 July 2023, J.G. Hill, thorny shrub in Chihuahuan Desert, Chianti Mountains. Deposited in the Mississippi Entomological Museum.

#### Specimens examined.

USA, **Texas**: • Presidio Co.; Chianti Mountains, 30 September 1928, E.R. Tinkham, (1♀) • 2.3 mi S Shafter, 23 July 1956, T.J. Cohn, B. Mathews (1♂) • Shafter cemetery, 29.8112, -104.3058, 28 September 2024, J.G. Hill, J.L. Seltzer (1♀).

#### Habitat.

Chihuahuan Desert Scrub (Fig. 33C) on leguminous shrubs and *Yucca*. Cohn (1956) describes the habitat at 2.3 mi S Shafter as “rocky foothills of the Chiantis on Spanish bayonet [*Yucca* sp.]”.

#### Distribution.

Known only from the Chianti Mountains of southwest Texas (Figs 25, 26A).

#### Etymology.

The species name *chiantiensis* is derived from the Chianti Mountains where the species is apparently endemic to and the suffix “-ensis” (Latin) meaning “originating from” or “inhabiting”. This name reflects the endemic nature of the species and hopefully draws attention to the importance of conservation of the unique biodiversity in this understudied mountainous region.

#### Suggested common name.

Chianti aridland scrub jumper.

### ﻿ Agroecotettix dorni sp. nov.

Taxon classificationAnimaliaOrthopteraAcrididae

AF722C9F-4F25-5326-AAB4-D43FE44275C7

https://zoobank.org/B2B38F60-80C4-4F45-8339-3A0C370D2655

**Figure 17.**
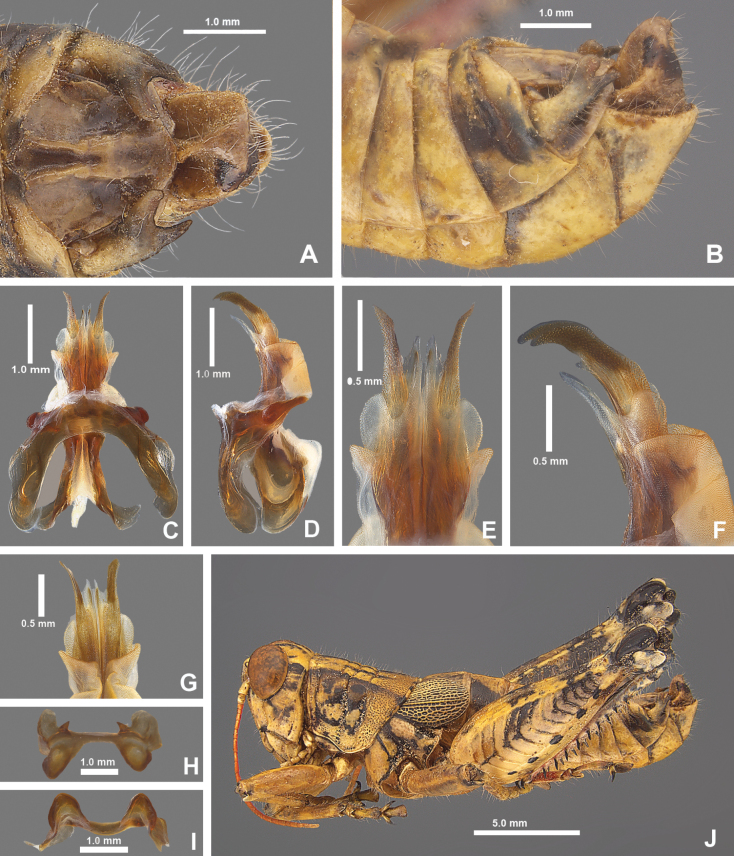
*Agroecotettixdorni***A** dorsal view of male terminalia **B** lateral view of male terminalia **C** dorsal view of phallic complex **D** lateral view of phallic complex **E** dorsal view of aedeagus **F** lateral view of aedeagus **G** caudal view of the aedeagus **H** dorsal view of epiphallus **I** caudal view of epiphallus **J** habitus.

Figs 2I , 4M , 5M , 17A–J , 25 , 26A , 34A–D

#### Diagnosis.

Differentiated from other species in the genus by the combination of male cerci with ventral branch equal or subequal in length to dorsal (Figs 2I, 17A, B); sheath of aedeagus thin and lightly sclerotized (Figs 5M, 17C–F); valves of the aedeagus are lobate, and in lateral view, the valves are thinly falcate, long and sword-like (Figs 5M, 17D, F), and in caudal view the lateral margins extend well beyond the rest of the valves and their apical margins are slightly curved distally (Figs 4M, 17G).

#### Male measurements (mm).

(*n* = 4) Body length 18.5–19.9 (mean = 19.2); pronotum length 4.1–4.5 (mean = 4.3); tegmen length 2.5–3.0 (mean = 2.8); hind femur length 10.7–11.0 (mean = 10.9); cerci length 1.2 (mean = 1.2); basal width of cercus 0.5–0.6 (mean = 0.6); mid-cercal width 0.4 (mean = 0.4); cerci dorsal fork length 0.3 (mean = 0.3); cerci dorsal fork apex width 0.3 (mean = 0.3) cerci ventral fork length 0.3 (mean = 0.3); cerci ventral fork apex width 0.2 (mean = 0.2).

#### Phallus measurements (mm).

(*n* = 2) Length 1.0 (mean = 1.0); apex width 0.4 (mean = 0.4); middle width 0.4 (mean = 0.4); Basal width 0.6–0.7 (mean = 0.7); lateral apex width 0.3 (mean = 0.3); lateral medial width 0.4 (mean = 0.4); lateral basal width 0.5 (mean = 0.5).

#### Female measurements (mm).

(*n* = 4) Body length 22.0–25.5 (mean = 24.1); pronotum length 5.1–6.0 (mean = 5.7) tegmen length 3.4–4.2 (mean = 3.8); hind femur length 12.2–15.0 (mean = 14.0); Dorsal ovipositor valve length 1.3–1.7 (mean = 1.5); ventral ovipositor valve length 1.3–1.7 (mean = 1.5).

#### Holotype.

• 1♂, USA, Texas, Brewster Co., Big Bend National Park, 29.1970, -102.9276, 14 July 2023, J.G. Hill, J.L. Seltzer; On shady side of sotol in mid-day heat, Boquillas Canyon. Deposited in the Mississippi Entomological Museum.

#### Specimens examined.

USA, **Texas**: • Brewster Co., Big Bend National Park, 29.1970, -102.9276, 14 July 2023, J.G. Hill, J.L. Seltzer (2♂, 4♀) • Big Bend National Park, Boquillas Ranger Station, 28–30 July 1956, T.J. Cohn and Mathews (1♀) • same data as previous, except 9 June 1961, T.J. and J.W. Cohn (1♀).

#### Habitat.

Chihuahuan Desert scrub (Fig. 33E). In July 2023 at the type locality, I observed this species roosting on the underside of *Dasylirion* leaves during the mid-day hours with *Netrosoma* and *Phaulotettix* species (Fig. 34A–D).

#### Distribution.

Endemic to the Chihuahuan Desert and more specifically to the southern big bend region of Texas (Figs 25, 26A). At present, all known populations occur within Big Bend National Park.

#### Etymology.

The species name *dorni* is a patronym honoring Michael Dorn, an American actor and narrator born in Texas who is most famous for portraying the Star Trek character Worf in the television series “Star Trek: The Next Generation” and its spin-offs. The name highlights a unique morphological characteristic of the species, drawing a creative parallel between the blade like aedeagus valves of the male genitalia and the form of the kur’leth, a traditional Klingon weapon used by Worf.

#### Suggested common name.

Dorn’s aridland scrub jumper.

### ﻿ Agroecotettix chisosensis sp. nov.

Taxon classificationAnimaliaOrthopteraAcrididae

E08F1D93-B9C3-5216-A81D-ADF979EC8122

https://zoobank.org/8E0B8267-E724-4B1B-8B73-71676E40E530

**Figure 18.**
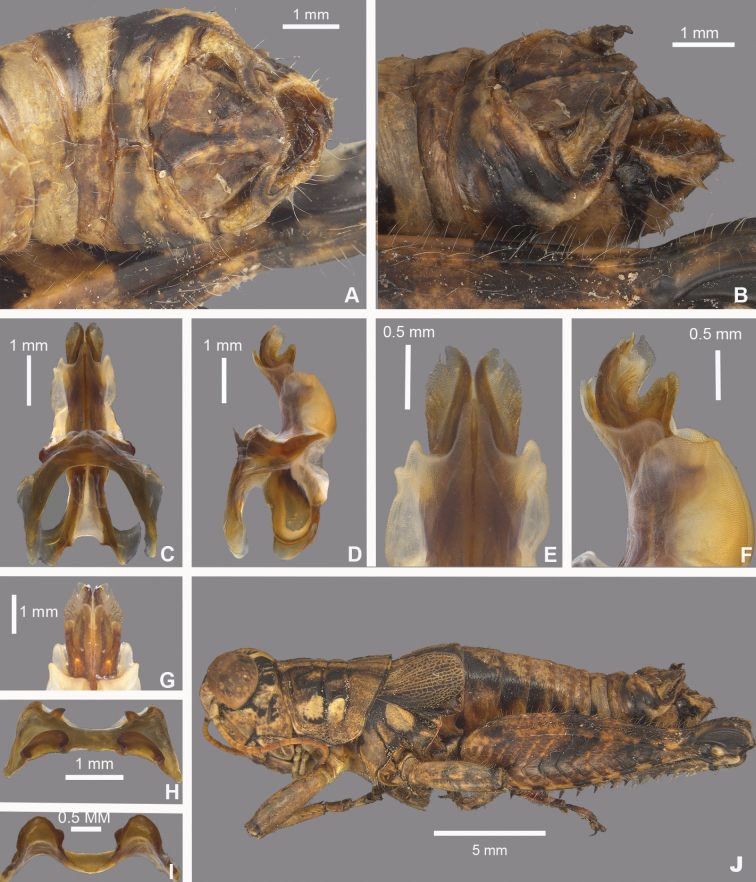
*Agroecotettixchisosensis***A** dorsal view of male terminalia **B** lateral view of male terminalia **C** dorsal view of phallic complex **D** lateral view of phallic complex **E** dorsal view of aedeagus **F** lateral view of aedeagus **G** caudal view of the aedeagus **H** dorsal view of epiphallus **I** caudal view of epiphallus **J** habitus.

Figs 2H , 4N , 5N , 18A–J , 25 , 26A , 36A–E

#### Diagnosis.

Differentiated from other species in the genus by the combination of male cerci with ventral branch equal or subequal in length to dorsal branch (Figs 2H, 18A, B); sheath of aedeagus thin and lightly sclerotized, (Fig. 5N); valves of the aedeagus are lobate, and in lateral view, the valves of aedeagus broad (Figs 5N, 18D, F) and in caudal view the lateral margins do not extend well beyond the rest of the valves and their apical margins are curved medially (Figs 4N, 18G).

#### Male measurements (mm).

(*n* = 8) Body length 18.7–21.3 (mean = 19.9); pronotum length 4.5–4.9 (mean = 4.6); tegmen length 3.0–3.6 (mean = 3.2); hind femur length 10.3–11.5 (mean = 10.8); cerci length 1.1–1.2 (mean = 1.2); basal width of cercus 0.6–0.7 (mean = 0.6); mid-cercal width 0.3–0.4 (mean = 0.4); cerci dorsal fork length 0.4–0.5 (mean = 0.4); cerci dorsal fork apex width 0.2 (mean = 0.2) cerci ventral fork length 0.3 (mean = 0.3); cerci ventral fork apex width 0.1 (mean = 0.1).

#### Phallus measurements (mm).

(*n* = 4) Length 0.9–1.1 (mean = 1.1); apex width 0.3–0.5 (mean = 0.4); middle width 0.4–0.6 (mean = 0.5); basal width 0.6 (mean = 0.6); lateral apex width 0.3–0.5 (mean = 0.4); lateral medial width 0.4–0.5 (mean = 0.4); lateral basal width 0.5 (mean = 0.5).

#### Female measurements (mm).

(*n* = 5) Body length 23.5–26.8 (mean = 25.1); pronotum length 5.5–6.2 (mean = 5.8) tegmen length 3.5–4.5 (mean = 4.0); hind femur length 12.9–13.9 (mean = 13.5); Dorsal ovipositor valve length 1.5–2.0 (mean = 1.8); ventral ovipositor valve length 1.5–2.0 (mean = 1.8).

#### Holotype.

• 1♂, USA, Texas, Brewster Co., Big Bend National Park, 29.2706, -103.3017, 14 July 2023, J.G. Hill; Chisos Mountain desert scrub, eating *Dasylirion* pollen. Deposited in the Mississippi Entomological Museum.

#### Specimens examined.

USA, **Texas**: • Brewster Co., Big Bend National Park, 29.2706, -103.3017, 14 July 2023, J.G. Hill (3♂, 1♀) • Juniper Canyon, Chisos Mts, 16 July 1928, F.M. Gaige (2♀) • Canyon behind Pulliam Bluff, Chisos Mts., 7 September 1912, Rehn and Hebard, 4000–5000 ft (2♂, 2♀) • Chisos Mts, 12 August 1940, Rehn and Hebard (1♂, 1♀) • Neville Springs, 8 September 1912, Rehn and Hebard (1♂).

#### Habitat.

Chihuahuan Desert scrub (Fig. 35F) in the Chisos Mountains. In July 2023 I observed the species eating *Dasylirion* pollen (Fig. 35D, E).

#### Distribution.

Endemic to the Chisos Mountains in the Big Bend region of Texas (Figs 25, 26A).

#### Etymology.

The species name *chisosensis* is derived from the Chisos Mountains where the species is apparently endemic to and the suffix “-ensis” (Latin) meaning “originating from” or “inhabiting”. This name reflects the endemic nature of the species and hopefully draws attention to the importance of conservation of the unique biodiversity in this mountainous region.

#### Suggested common name.

Chisos aridland scrub jumper.

### ﻿ Agroecotettix turneri sp. nov.

Taxon classificationAnimaliaOrthopteraAcrididae

09F0B082-EF6D-5E7A-B808-C86A0E1B0DC5

https://zoobank.org/C09F938B-B081-42DD-A82D-7A1A193957C9

**Figure 19.**
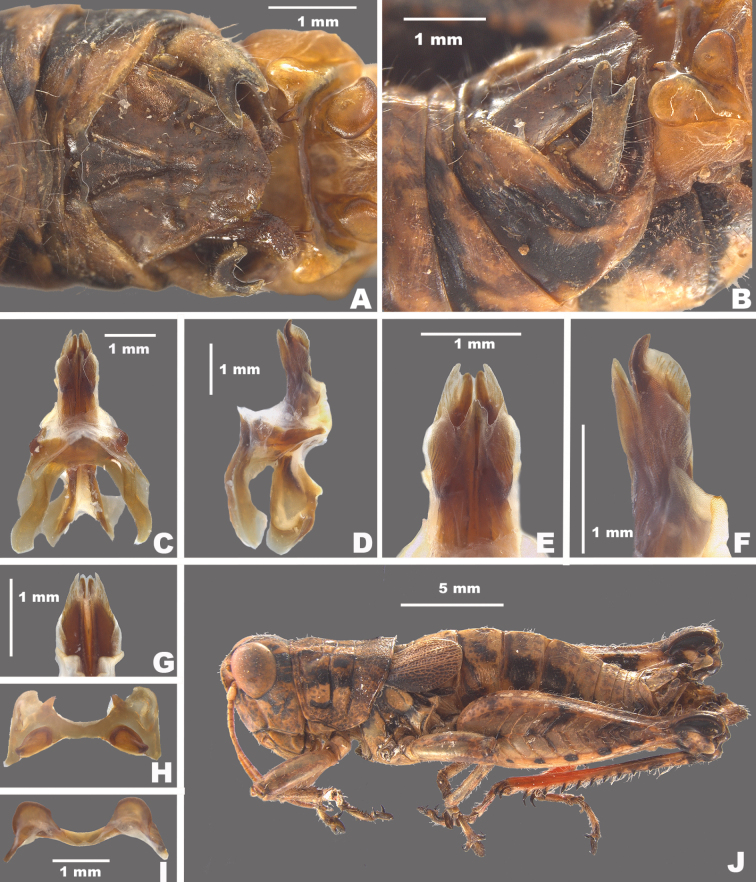
*Agroecotettixturneri***A** dorsal view of male terminalia **B** lateral view of male terminalia **C** dorsal view of phallic complex **D** lateral view of phallic complex **E** dorsal view of aedeagus **F** lateral view of aedeagus **G** caudal view of the aedeagus **H** dorsal view of epiphallus **I** caudal view of epiphallus **J** habitus.

Figs 2K , 4K , 5K , 19A–J , 25 , 26A , 36A–D

#### Diagnosis.

Differentiated from other species in the genus by the combination of male cerci with ventral branch equal or subequal in length to dorsal branch (Figs 2K, 19A, B); sheath of aedeagus thin and lightly sclerotized (Fig. 5M), and in lateral view, the valves of the aedeagus are entire (not lobate) with apices of point caudally (Figs 5K, 19D, F). In caudal view, lateral margins converge medially, giving a more pointed appearance (Figs 4N, 18G).

#### Male measurements (mm).

(*n* = 3) Body length 14.3–19.3 (mean = 17.7); pronotum length 3.2–4.5 (mean = 4.0); tegmen length 2.3–4.5 (mean = 4.0); hind femur length 7.9–10.7 (mean = 9.8); cerci length 0.8–1.2 (mean = 9.8); basal width of cercus 0.5–0.6 (mean = 0.6); mid-cercal width 0.3–0.4 (mean = 0.4); cerci dorsal fork length 0.3 (mean = 0.3); cerci dorsal fork apex width 0.1 (mean = 0.1) cerci ventral fork length 0.3–0.4 (mean = 0); cerci ventral fork apex width 0.2 (mean = 0.2).

#### Phallus measurements (mm).

(*n* = 3) Length 1.1 (mean = 1.1); apex width 0.3 (mean = 0.3); middle width 0.6 (mean = 0.6); Basal width 0.5–0.6 (mean = 0.6); lateral apex width 0.4 (mean = 0.4); lateral medial width 0.4 (mean = 0.4); lateral basal width 0.5 (mean = 0.5).

#### Female measurements (mm).

(*n* = 6) Body length 17.1–26.5 (mean = 23.2); pronotum length 4.4–6.5 (mean = 5.6) tegmen length 2.2–4.4 (mean = 3.7); hind femur length 9.7–15.0 (mean = 12.9); Dorsal ovipositor valve length 0.9–2.5 (mean = 1.7); ventral ovipositor valve length 0.9–2.1 (mean = 1.5).

#### Holotype.

• 1♂, USA, Texas, Brewster Co., 22 mi S of Alpine, 30.045026, -103.573517, 15 July 2023, J.G. Hill; Collected in Chihuahuan Desert scrub. Deposited in the Mississippi Entomological Museum.

#### Specimens examined.

USA, **Texas**: • Brewster Co., Alpine, 21 August 1939, F.B. Isely (1♂, 4♀) • 22 mi S of Alpine, 30.045026, -103.573517, 15 July 2023, J.G. Hill (1♀) • “Big Bend” 23 June 1947, R.H. Beamer (1♂, 1♀).

#### Habitat.

Chihuahuan Desert scrub on *Vachellia* sp. (Fig. 36D).

#### Distribution.

Found in the area between Big Bend National Park and Alpine, Texas (Figs 25, 26A).

#### Etymology.

The species name *turneri* is a patronym honoring Robert Edward “Ted” Turner III, an American media mogul and philanthropist renowned for his extensive contributions to environmental conservation. Turner, the founder of CNN and a major philanthropist, has been instrumental in numerous initiatives aimed at protecting the environment and biodiversity. His establishment of the Turner Endangered Species Fund and his efforts in large-scale land conservation have provided critical support for the preservation of diverse ecosystems, including those that likely sustain a great diversity of grasshopper species.

#### Suggested common name.

Turner’s aridland scrub jumper.

### ﻿ Agroecotettix quitmanensis sp. nov.

Taxon classificationAnimaliaOrthopteraAcrididae

127F4ED9-34CB-528D-BDAE-73C54FD4A5F9

https://zoobank.org/8A91A6D1-09C0-4758-A4C7-C6B36C77ECEE

**Figure 20.**
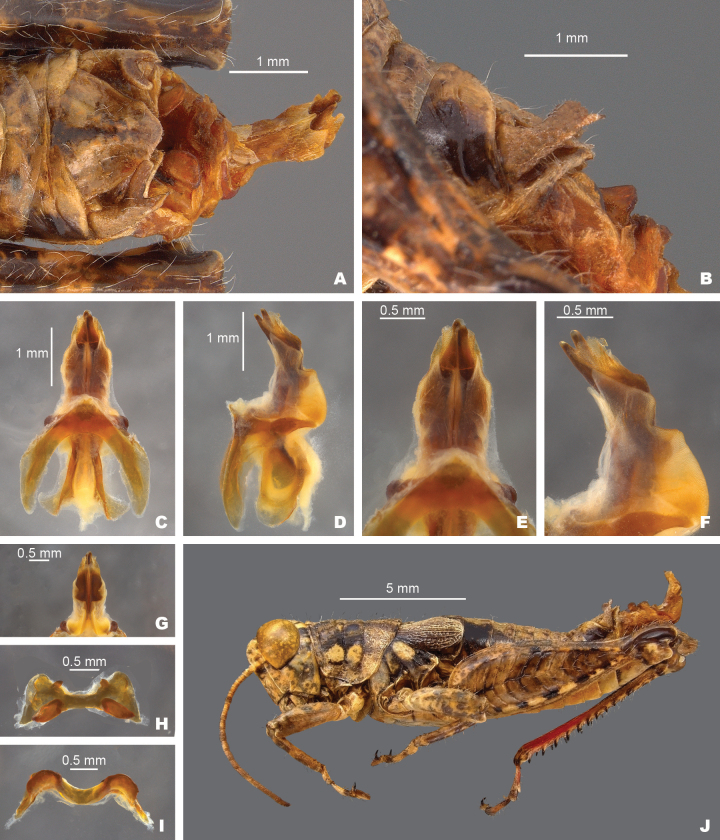
*Agroecotettixquitmanensis***A** dorsal view of male terminalia **B** lateral view of male terminalia **C** dorsal view of phallic complex **D** lateral view of phallic complex **E** dorsal view of aedeagus **F** lateral view of aedeagus **G** caudal view of the aedeagus **H** dorsal view of epiphallus **I** caudal view of epiphallus **J** habitus.

Figs 2L , 4P , 5P , 20A–J , 25 , 26A

#### Diagnosis.

Differentiated from other species in the genus by the combination of male cerci with ventral branch equal or subequal in length to dorsal branch as in Fig. 2; sheath of aedeagus thin and lightly sclerotized, as in Fig. 5L. In lateral view, the apical edge of the valves of the aedeagus are thickened finger-like projections that curve apically as in Fig. 5L.

#### Male measurements (mm).

(*n* = 1) Body length 17.4; pronotum length 4.0; tegmen length 2.7; hind femur length 9.7; cerci length 1.2; basal width of cercus 0.6; mid-cercal width 0.4; cerci dorsal fork length 0.4; cerci dorsal fork apex width 0.3; cerci ventral fork length 0.4; cerci ventral fork apex width 0.1.

#### Phallus measurements (mm).

(*n* = 1) Length 1.2; apex width 0.4; middle width 0.6; basal width 0.8; lateral apex width 0.2; lateral medial width 0.4; lateral basal width 0.4.

#### Female measurements (mm).

(*n* = 1) Body length 20.6; pronotum length 3.0; tegmen length 3.8; hind femur length 11.7; Dorsal ovipositor valve length 1.6; ventral ovipositor valve length 1.6.

#### Holotype examined.

• 1♂, USA, Texas, Quitman Mountains, El Paso Co., Sept. 14, 1912, H.[ebard], 4800–5100 ft. Deposited in the Mississippi Entomological Museum.

#### Habitat.

Unknown, but likely desert scrub as other species of the genus.

#### Distribution.

Found in the vicinity of the Quitman Mountains of southwest Texas (Fig. 25A).

#### Etymology.

The species name *quitmanensis* is derived from the Quitman Mountains where the species is apparently endemic to and the suffix “-ensis” (Latin) meaning “originating from” or “inhabiting”. This name reflects the endemic nature of the species and hopefully draws attention to the importance of conservation of the unique biodiversity in this understudied mountainous region.

#### Suggested common name.

Quitman aridland scrub jumper.

### ﻿ Agroecotettix vaquero sp. nov.

Taxon classificationAnimaliaOrthopteraAcrididae

C1920189-71C7-5C14-AFEA-E51730C2EAD5

https://zoobank.org/FA1E3188-352E-4EEB-966E-02FBF0C56D0E

**Figure 21.**
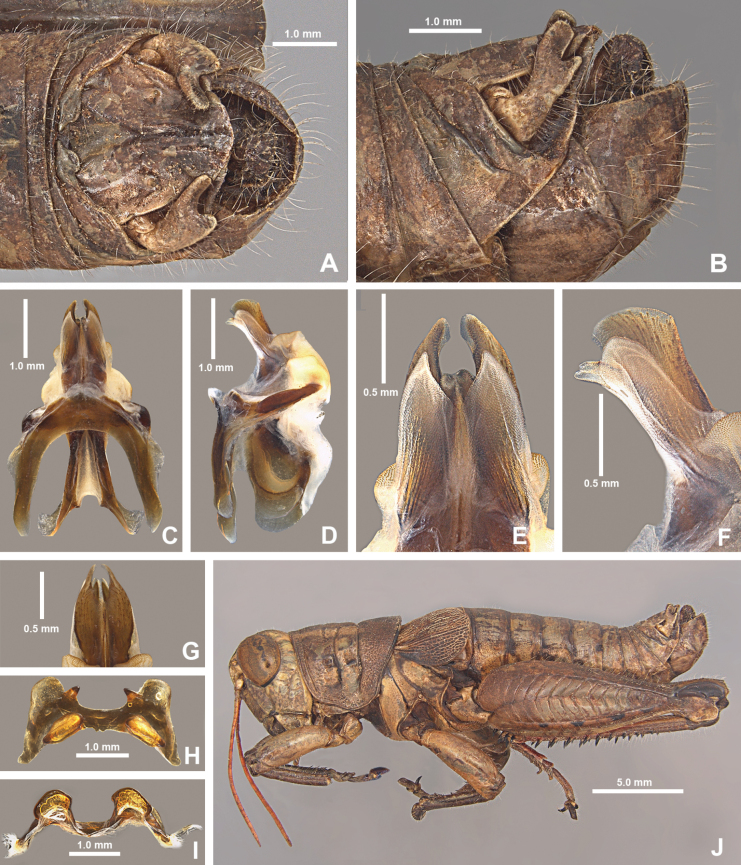
*Agroecotettixvaquero***A** dorsal view of male terminalia **B** lateral view of male terminalia **C** dorsal view of phallic complex **D** lateral view of phallic complex **E** dorsal view of aedeagus **F** lateral view of aedeagus **G** caudal view of the aedeagus **H** dorsal view of epiphallus **I** caudal view of epiphallus **J** habitus.

Figs 2T , 4P , 5P , 21A–J , 25

#### Diagnosis.

Differentiated from other species in the genus by the combination of male cerci with the ventral branch reduced and rounded as in Fig. 2S; in lateral view, the sheath of the aedeagus is well developed and expanded laterally around the valves; the aedeagus valves are wide with their apices broadly curved in lateral view as in Fig. 5P; in caudal view the valves or greatly narrowed in their apical third as in Fig. 4P.

#### Male measurements (mm).

(*n* = 17) Body length 21.2–25.2 (mean = 23.5); pronotum length 4.7–6.0 (mean = 5.4); tegmen length 2.7–4.1 (mean = 3.4); hind femur length 10.9–13.2 (mean = 12.1); cerci length 1.0–1.7 (mean = 1.3); basal width of cercus 0.5–0.7 (mean = 0.6); mid-cercal width 0.4–0.6 (mean = 0.5); cerci dorsal fork length 0.4–0.6 (mean = 0.5); cerci dorsal fork apex width 0.2–0.4 (mean = 0.3); cerci ventral fork length 0.1–0.3 (mean = 0.2); cerci ventral fork apex width 0.1 (mean = 0.1).

#### Phallus measurements (mm).

(*n* = 4) Length 0.8–1.0 (mean = 1.0); apex width 0.3 (mean = 0.3); middle width 0.5 (mean = 0.5); basal width 0.6–0.7 (mean = 0.6); lateral apex width0.3–0.4 (mean = 0.4); lateral medial width 0.5–0.6 (mean = 0.5); lateral basal width 0.5 (mean = 0.5).

#### Female measurements (mm).

(*n* = 17) Body length 24.5–29.9 (mean = 26.6); pronotum length 5.9–7.2 (mean = 6.5) tegmen length 3.1–4.8 (mean = 3.9); hind femur length 13.0–15.4 (mean = 14.3); dorsal ovipositor valve length 1.3–2.2 (mean = 1.7); ventral ovipositor valve length 1.2–2.2 (mean = 1.7).

#### Holotype.

• 1♂, Mexico, Coahulla,11 mi NW Muzquiz, 31 July 1959, 1550 ft, T.J. Cohn, #126, UMMZI-0058033. Deposited in the Mississippi Entomological Museum.

#### Specimens examined.

Mexico: **Coahuila**: • 5.8 mi S Castaños, 20 August 1965, T.J. Cohn, 2700 ft, (3♂, 1♀) • 2 mi NW Hermanas, 19 September 1958 13–1400’ T.J. Cohn (1♂) • 5 mi S Hermanas, 1 August 1959, T.J. Cohn, 1350 ft (1♂, 1♀) • 2 mi SE Muzquiz, 14 September 1958, T.J. Cohn, 3700 ft (3♂, 5♀) • 4 mi E Muzquiz, 1 August 1959, T.J. Cohn, 1600 ft (2♂, 2♀) • 2 mi SE Muzquiz, 14 September 1958, T.J. Cohn, 3700 ft. (2♂, 2♀) • 11 mi NW Muzquiz, 31 July 1959, T.J. Cohn, 1550 ft (3♂, 2♀) • San Juan de Sabinas-Rosita, 15–16 September 1958, T.J. Cohn, 1200 ft (5♂, 4♀).

#### Habitat.

Cohn (1965) described the habitat at 5.8 mi S Castanos as low Mesquite, Creosote, *Vachelliarigidula*, and *Agavelechuguilla*.

#### Distribution.

Found in northern Coahuila, Mexico (Fig. 25A).

#### Etymology.

The species name *vaquero* is the Spanish word for cowboy.

#### Suggested common name.

Vaquero aridland scrub jumper.

### ﻿ Agroecotettix forcipatus sp. nov.

Taxon classificationAnimaliaOrthopteraAcrididae

24CEC121-EF01-5BB1-AE16-F4C0051E2FB2

https://zoobank.org/97EE7BEC-3C6A-44E2-B54D-EF09C9727251

**Figure 22.**
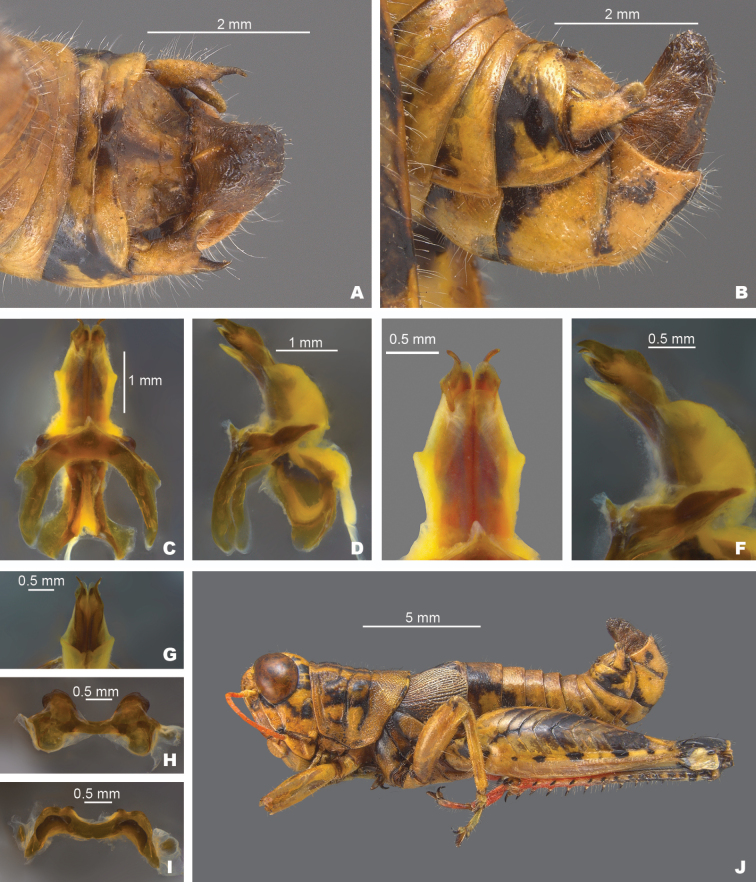
*Agroecotettixforcipatus***A** dorsal view of male terminalia **B** lateral view of male terminalia **C** dorsal view of phallic complex **D** lateral view of phallic complex **E** dorsal view of aedeagus **F** lateral view of aedeagus **G** caudal view of the aedeagus **H** dorsal view of epiphallus **I** caudal view of epiphallus **J** habitus.

Figs 2O , 4O , 5O , 22A–J , 25

#### Diagnosis.

Differentiated from other species in the genus by the combination of male cerci with dorsal and ventral branches that are short but equal or subequal in length and widely separated as in Fig. 2N; sheath of aedeagus thin and lightly sclerotized (Fig. 5O); in lateral view, the valves of the aedeagus are acutely pointed apically and are greatly widened in their lower half; in caudal view the apical margins of the valves are slightly curved distally as in Fig. 4O.

#### Male measurements (mm).

(*n* = 12) Body length 21.0–23.5 (mean = 22.1); pronotum length 4.3–5.5 (mean = 4.8); tegmen length 2.5–4.1 (mean = 3.3); hind femur length 10.6–12.2 (mean = 11.4); cerci length 1.2–1.5 (mean = 1.3); basal width of cercus 0.6–0.8 (mean = 0.7); mid-cercal width 0.3–0.5 (mean = 0.4); cerci dorsal fork length 0.3–0.4 (mean = 0.4); cerci dorsal fork apex width 0.2–0.3 (mean = 0.3); cerci ventral fork length 0.3–0.5 (mean = 0.4); cerci ventral fork apex width 0.1 (mean = 0.1).

#### Phallus measurements (mm).

(*n* = 4) Length 1.3–1.4 (mean = 1.4); apex width 0.4–0.5 (mean = 0.5); middle width 0.5–0.6 (mean = 0.5); basal width 0.9–1.0 (mean = 1.0); lateral apex width 0.3–0.4 (mean = 0.3); lateral medial width 0.6 (mean = 0.6); lateral basal width 0.6–0.8 (mean = 0.7).

#### Female measurements (mm).

(*n* = 10) Body length 24.0–27.5 (mean = 25.2); pronotum length 5.1–6.2 (mean = 5.6) tegmen length 3.5–4.2 (mean = 3.8); hind femur length 12.0–14.2 (mean = 13.3); dorsal ovipositor valve length 1.5–2.0 (mean = 1.6); ventral ovipositor valve length 1.5–2.0 (mean = 1.6).

#### Holotype.

• 1♂, Mexico, Coahuila, 22.6 mi S Castaños, (11.2 mi N of San Lazaro), 19 August 1961, I.J. Cantrall, T.J. Cohn. UMMZI-00057993. Deposited in the Mississippi Entomological Museum.

#### Specimens examined.

Mexico, **Coahuila**: • 25 mi S Castaños, 3 August 1959, T.J. Cohn 3150 ft (8♂, 9♀) • 5 mi S Monolova, 2 August 1959, T.J. Cohn, 2300 ft (6♂, 2♀).

#### Habitat.

Cohn (1959) described the habitat at 5 mi S. Monolova as rich lush desert, with very little grass consisting of a few species, but many types of succulent stem bushes in abundance along with leguminous bushes and broad leaf black berries.

#### Distribution.

Found southern Coahuila, Mexico in the vicinity of the Sierra de la Gloria (Fig. 25A)

#### Etymology.

*forceps* Latin = forceps, pincers and *atus* Latin = “provided with”.

#### Suggested common name.

Pincered aridland scrub jumper.

### ﻿ Agroecotettix idic sp. nov.

Taxon classificationAnimaliaOrthopteraAcrididae

4BA10222-3118-5BC4-81E5-C9B84337FECE

https://zoobank.org/D4BF1146-CC49-4EFB-8C3A-C30C111200B5

**Figure 23.**
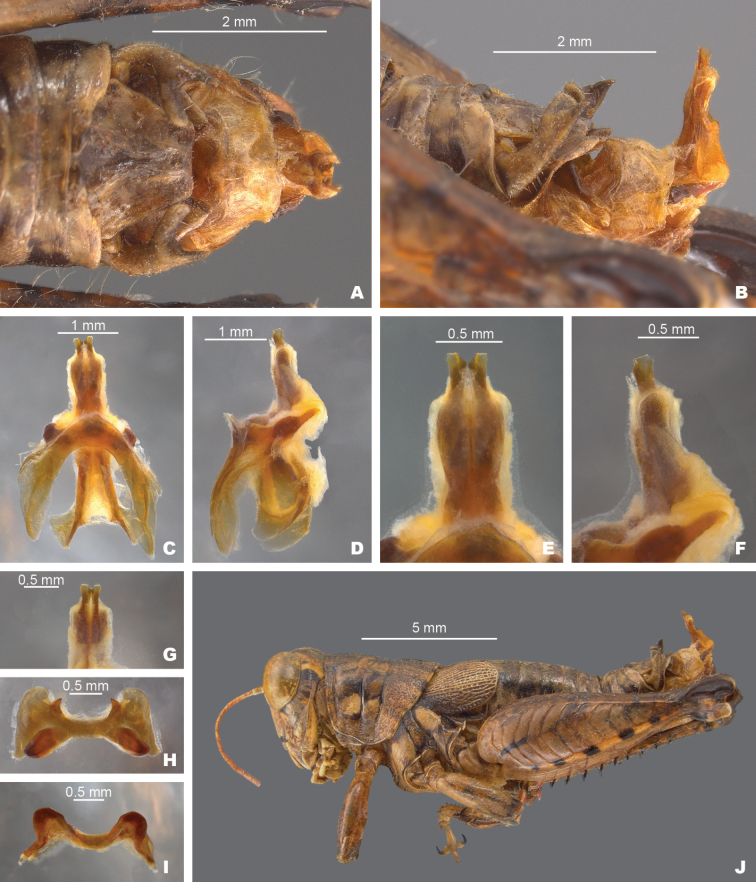
*Agroecotettixidic***A** dorsal view of male terminalia **B** lateral view of male terminalia **C** dorsal view of phallic complex **D** lateral view of phallic complex **E** dorsal view of aedeagus **F** lateral view of aedeagus **G** caudal view of the aedeagus **H** dorsal view of epiphallus **I** caudal view of epiphallus **J** habitus.

Figs 2S , 4Q , 5Q , 23A–J , 25 , 26B

#### Diagnosis.

Differentiated from other species in the genus by the combination of male cerci that curve medially (Fig. 2Q) and with dorsal and ventral branches that are short but equal or subequal in length and widely separated as in Fig. 2R; in lateral view, the sheath of the aedeagus is thin and shorter than the valves; the aedeagus valves are the most diminutive of the genus and are rectangular in shape, the distal edge is edge truncate in lateral (Fig. 4Q) and in caudal view (Fig. 5Q).

#### Male measurements (mm).

(*n* = 1) Body length 18.5; pronotum length 4.4; tegmen length 3.0; hind femur length 10.0; cerci length 1.5; basal width of cercus 0.6; mid-cercal width 0.5; cerci dorsal fork length 0.3; cerci dorsal fork apex width 0.3; cerci ventral fork length 0.5; cerci ventral fork apex width 0.1.

#### Phallus measurements (mm).

(*n* = 1) Length 1.5; apex width 0.2; middle width 0.5; basal width 0.4; lateral apex width 0.2; lateral medial width 0.3; lateral basal width 0.4.

#### Female measurements (mm).

(*n* = 1) Body length 24.2; pronotum length 5.7; tegmen length 3.5; hind femur length 12.5; dorsal ovipositor valve length 2.0; ventral ovipositor valve length 2.0.

#### Holotype.

• 1♂, Mexico, Higueros, Coah., Mex. Bet. Monterrey and Saltillo, 4000’, IX 14 1936, H.R. Roberts. Deposited in the Mississippi Entomological Museum.

#### Habitat.

Unknown, but likely desert scrub as other species of the genus.

#### Distribution.

Known only from the type locality (Fig. 25A, C).

#### Etymology.

The species epithet *idic* references the IDIC principle from the Star Trek television series. IDIC stands for “Infinite Diversity in Infinite Combinations,” a Vulcan philosophy celebrating the richness and complexity of the universe. This name pays homage to the Star Trek principle of embracing diversity and complexity and highlights the rich biodiversity found in Mexico, the native land of this grasshopper. It is hoped that this name encourages appreciation and protection of the diverse forms of life that coexist on our planet.

#### Suggested common name.

Idic aridland scrub jumper.

### ﻿ Agroecotettix kahloae sp. nov.

Taxon classificationAnimaliaOrthopteraAcrididae

771CE4E2-3FB2-5B6D-A999-4F6E1CE9D0F5

https://zoobank.org/EAA95587-C4F7-4E95-83B3-42B6EFD80515

**Figure 24.**
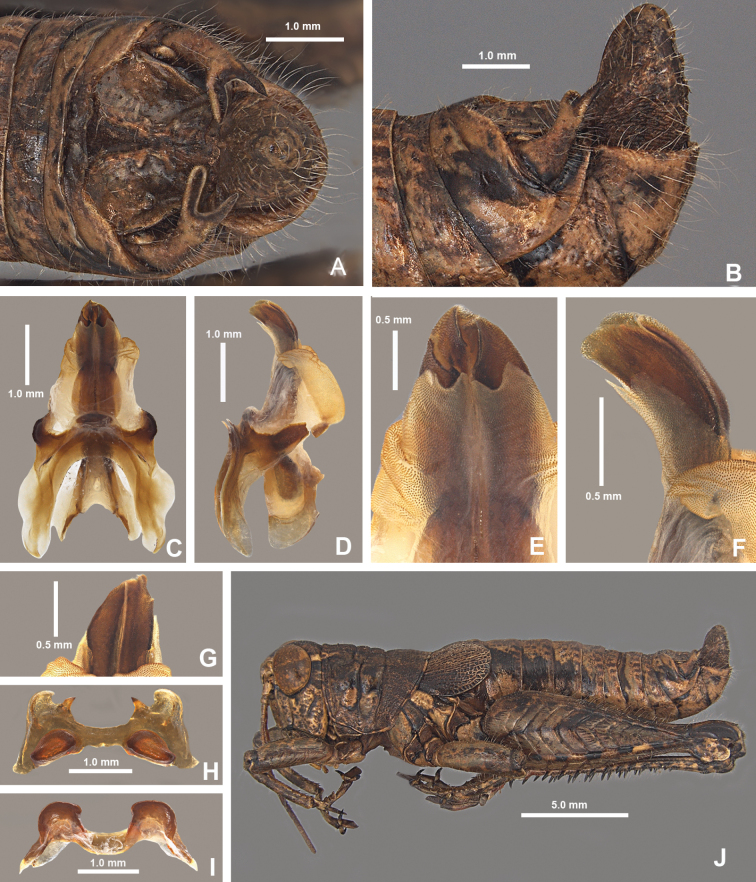
*Agroecotettixkahloae***A** dorsal view of male terminalia **B** lateral view of male terminalia **C** dorsal view of phallic complex **D** lateral view of phallic complex **E** dorsal view of aedeagus **F** lateral view of aedeagus **G** caudal view of the aedeagus **H** dorsal view of epiphallus **I** caudal view of epiphallus **J** habitus.

**Figure 25.**
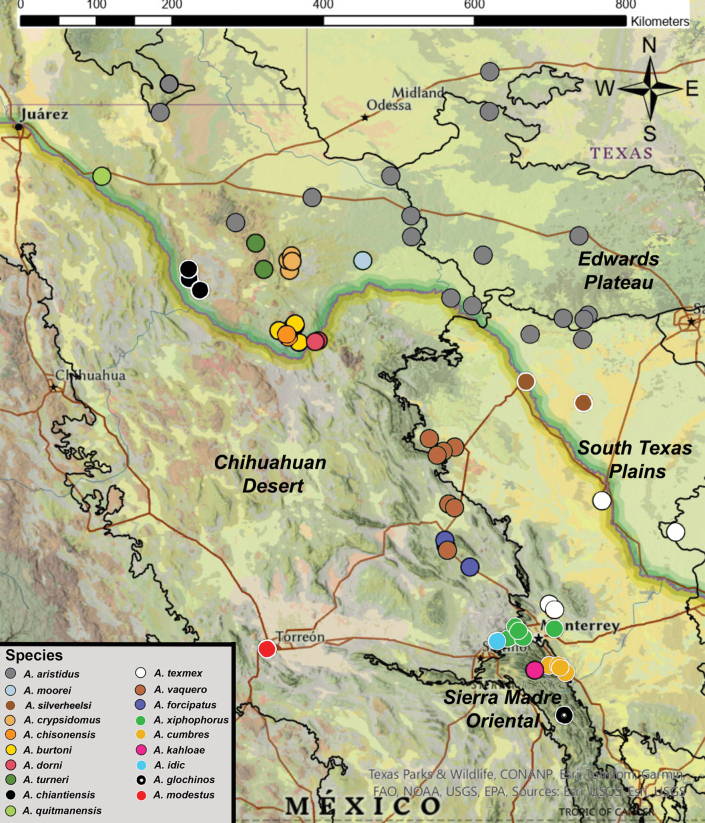
Distribution of *Agroecotettix* species.

**Figure 26.**
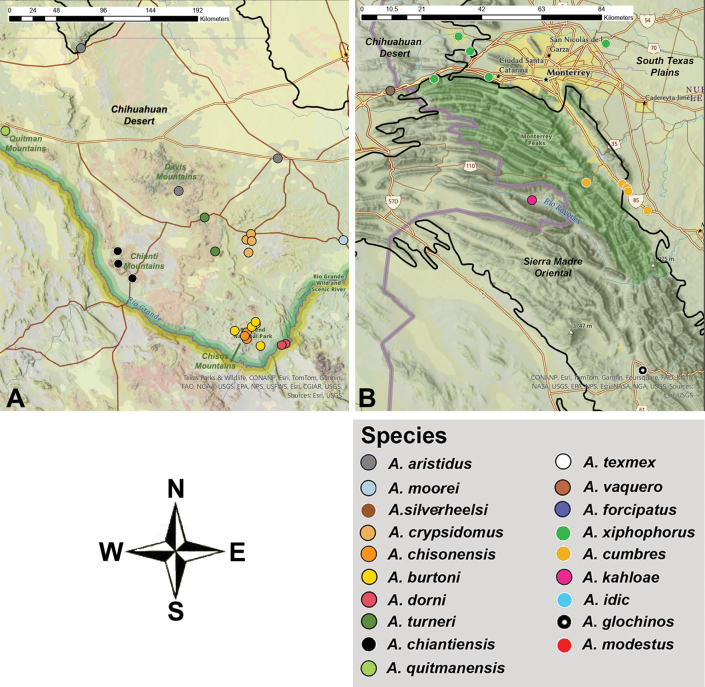
Distribution of *Agroecotettix* species **A** Big Bend region of Texas **B** zoom in on the area around Monterrey, Mexico at the convergence of the Chihuahuan Desert, Sierra Madre Oriental, and South Texas Plains ecoregions.

Figs 2Q , 4R , 5R , 24A–J , 25 , 26B

#### Diagnosis.

Differentiated from other species in the genus by the combination of male cerci that strongly curve medially as in Fig. 2Q, R; and with dorsal and ventral branches that are short but equal or subequal in length and widely separated (Fig. 2R); sheath of aedeagus thin and lightly sclerotized, as in Fig. 5R; in lateral view the valves of the aedeagus are broad and arching with the distal apices rounded as in Fig. 5R; in caudal view the valves are acuminate.

#### Male measurements (mm).

(*n* = 1) Body length 18.5; pronotum length 4.2; tegmen length 2.7; hind femur length 9.9; cerci length 1.3; basal width of cercus 0.4; mid-cercal width 0.5; cerci dorsal fork length 0.4; cerci dorsal fork apex width 0.2; cerci ventral fork length 0.5; cerci ventral fork apex width 0.1.

#### Phallus measurements (mm).

(*n* = 1) Length 1.2; apex width 0.2; middle width 0.5; basal width 0.8; lateral apex width 0.3; lateral medial width 0.4; lateral basal width 0.6.

#### Holotype.

• 1♂, Mexico, Coahuila, 29 rd, mi SE Arteaga, 10 August 1959, 6150 ft., T.J. Cohn, #164. Deposited in the Mississippi Entomological Museum.

#### Habitat.

None recorded.

#### Distribution.

Known only from the type locality (Fig. 25A, C).

**Figure 27.**
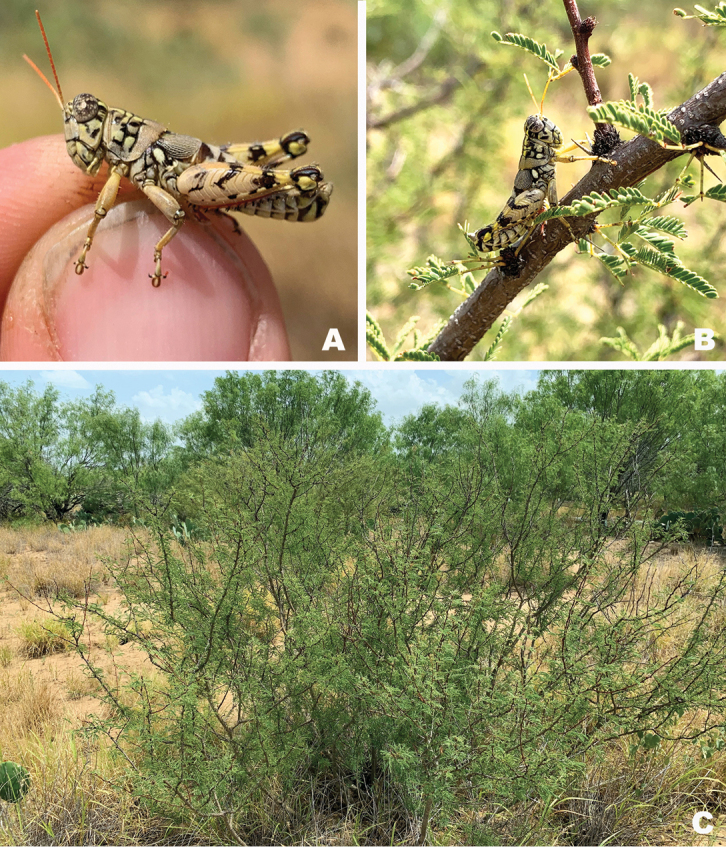
*Agroecotettixsilverheelsi* from Dimmit Co, TX **A** male **B** male on interior *Vachellia* branch **C***Vachellia* plant and habitat where the type specimen was collected.

**Figure 28.**
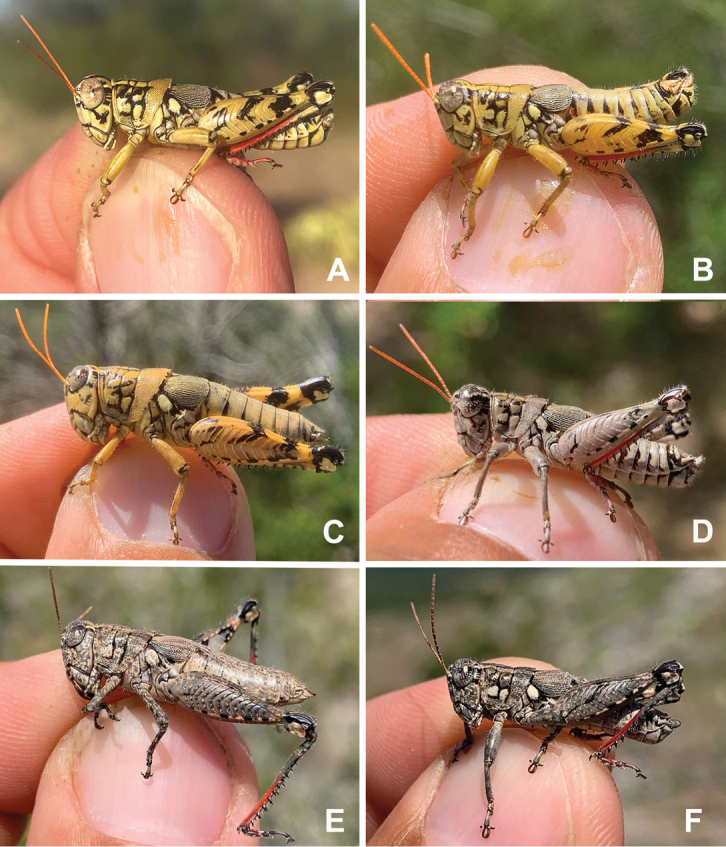
*Agroecotettixaristus***A** male, Upton Co, TX **B** male, Edwards Co., TX **C** female, Edwards Co., TX **D** male Edwards Co., TX **E** female, Edwards Co., TX **F** male, Uvalde, Co., TX.

**Figure 29.**
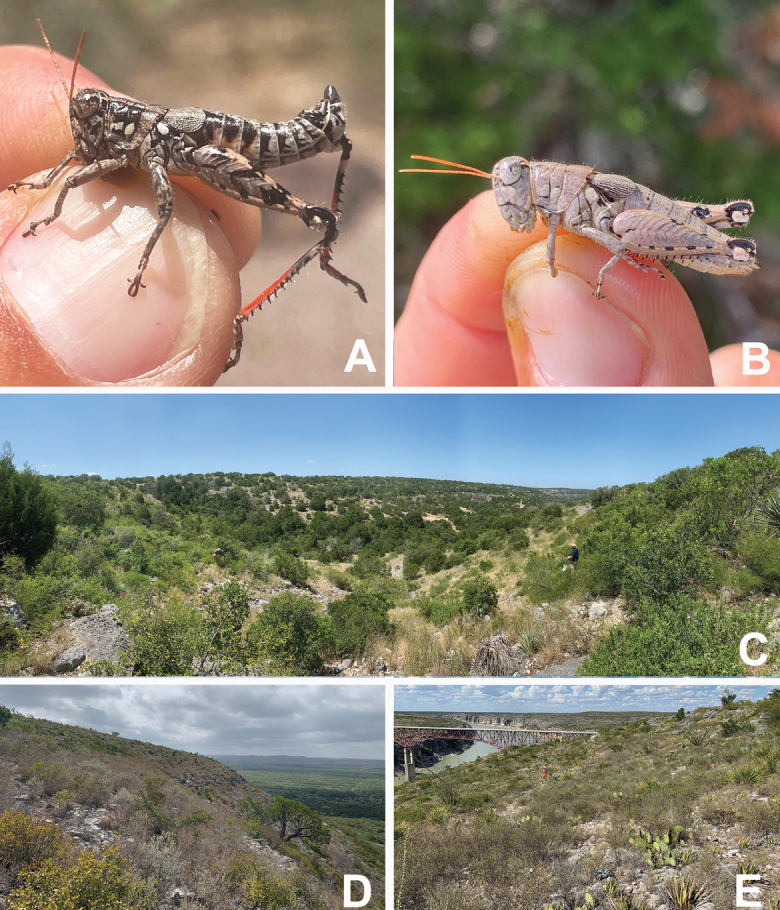
*Agroecotettixaristus***A** male, Jeff Davis Co., TX **B** female Edwards Co., TX **C** habitat in Edwards Co., TX **D** habitat in Uvalde Co, TX **E** habitat in Val Verde Co., TX.

**Figure 30.**
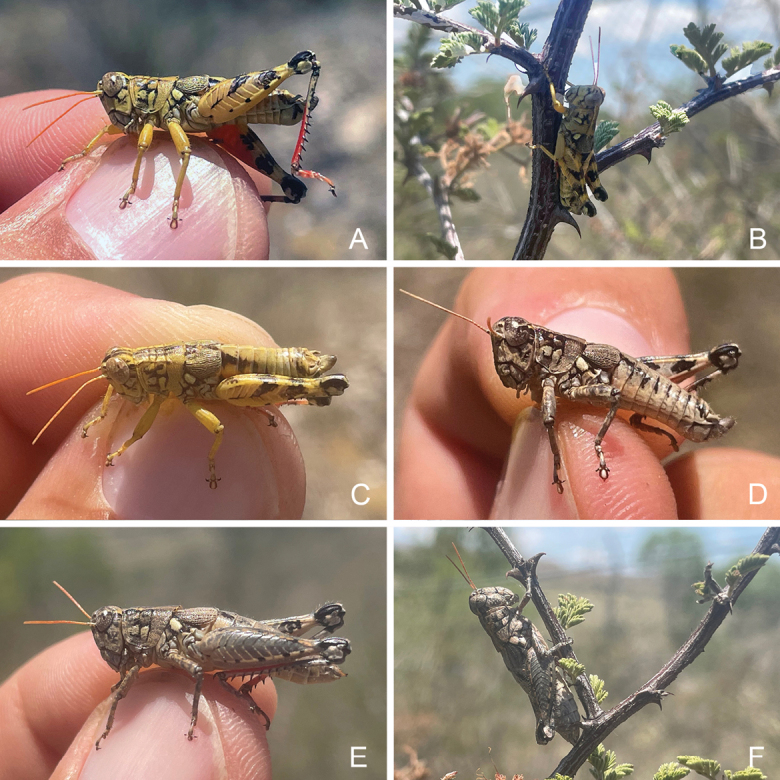
*Agroecotettixcrypsidomus* from Brewster Co., Texas **A** male **B** male on *Vachellia***C** male **D** male **E** female **F** female on *Vachellia*.

**Figure 31.**
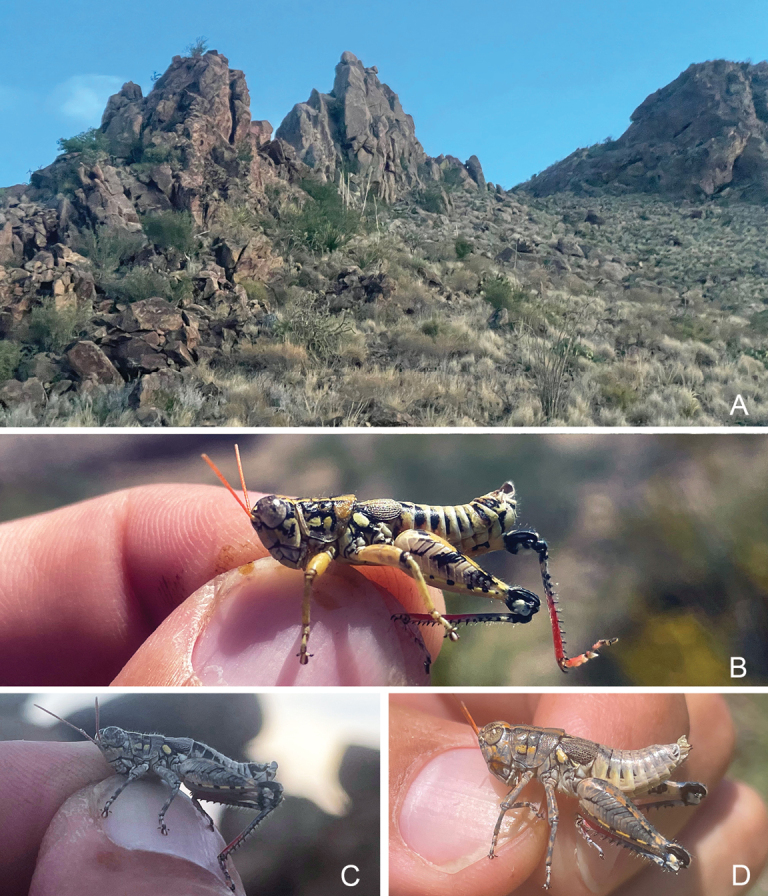
*Agroecotettixburtoni* from Brewster Co., Texas **A** habitat view in Big Bend National Park **B** male **C** male **D** male.

**Figure 32.**
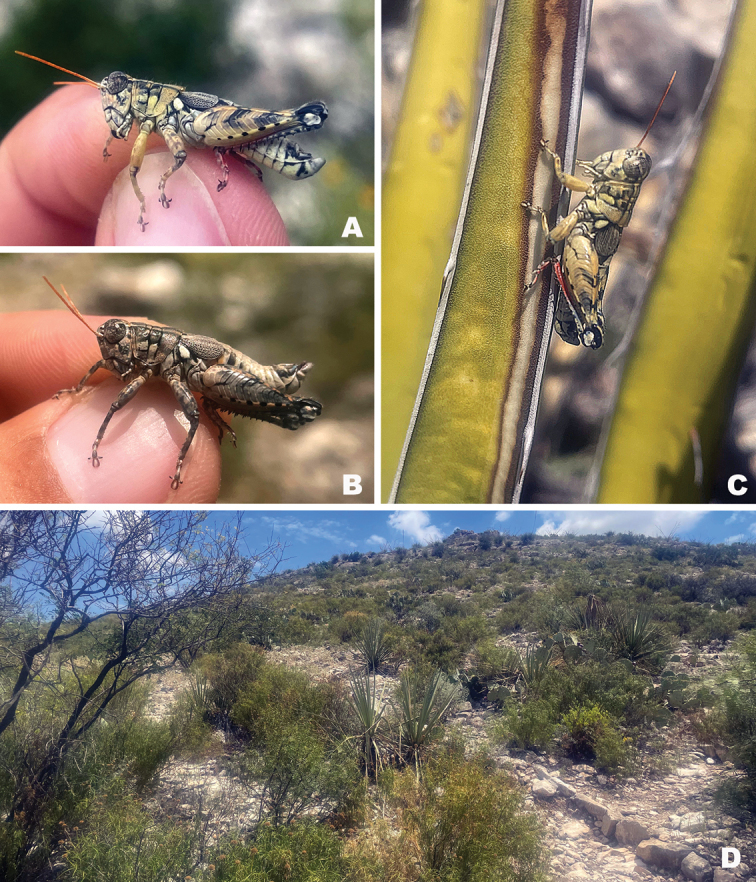
*Agroecotettixmoorei* Terrel Co., Texas **A** male **B** male **C** male **D** habitat view near Sanderson, TX.

**Figure 33.**
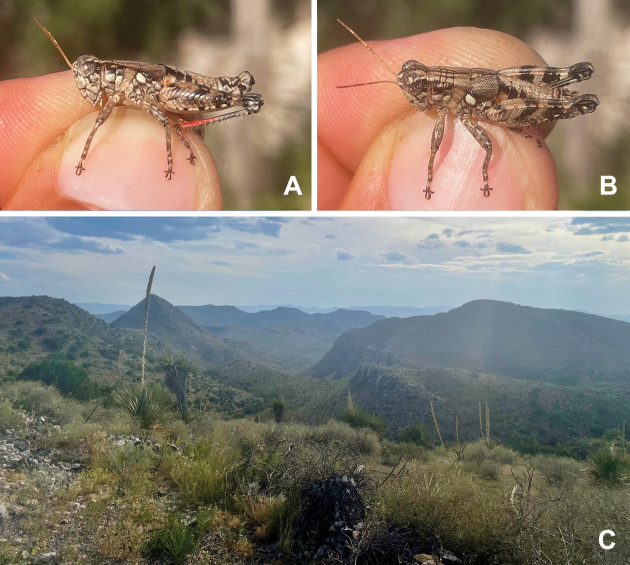
*Agroecotettixchiantiensis* from Presidio Co., Texas **A** male in lateral view **B** male is semi-dorsal view **C** habitat view.

**Figure 34.**
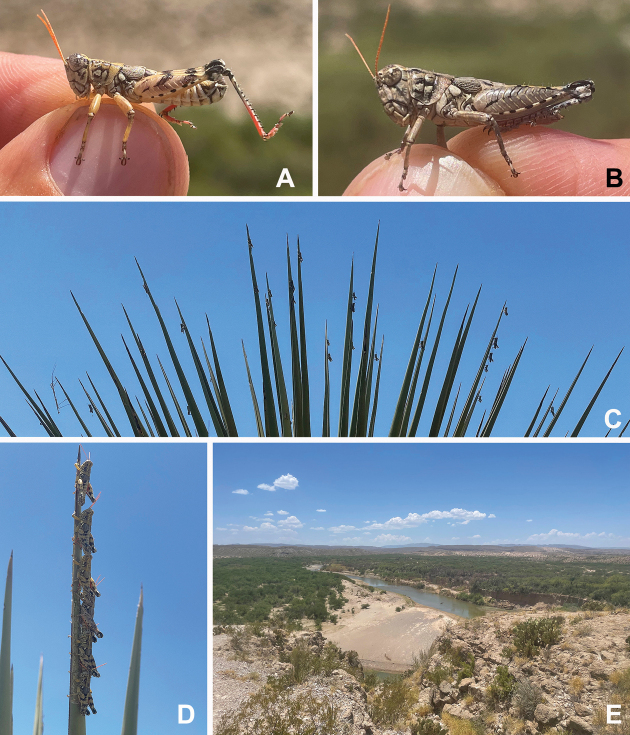
*Agroecotettixdorni* from Brewster Co., Texas **A** male in lateral view **B** female in lateral view **C** Individuals roosting during the heat of the day on *Dasylirion* leaves **D** habitat view.

**Figure 35.**
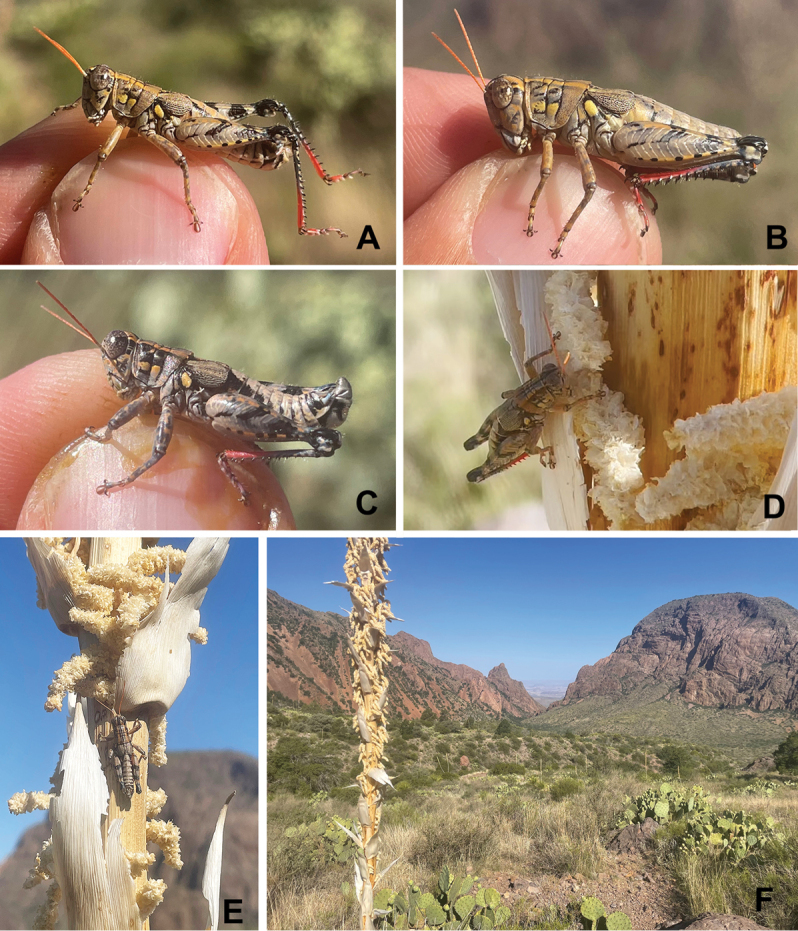
*Agroecotettixchisosensis* from Brewster Co., Texas **A** male in lateral view **B** female in lateral view **C** male in lateral view **D** male feeding on *Dasylirion* flowers **E** male feeding on *Dasylirion* flowers **F** habitat view.

**Figure 36.**
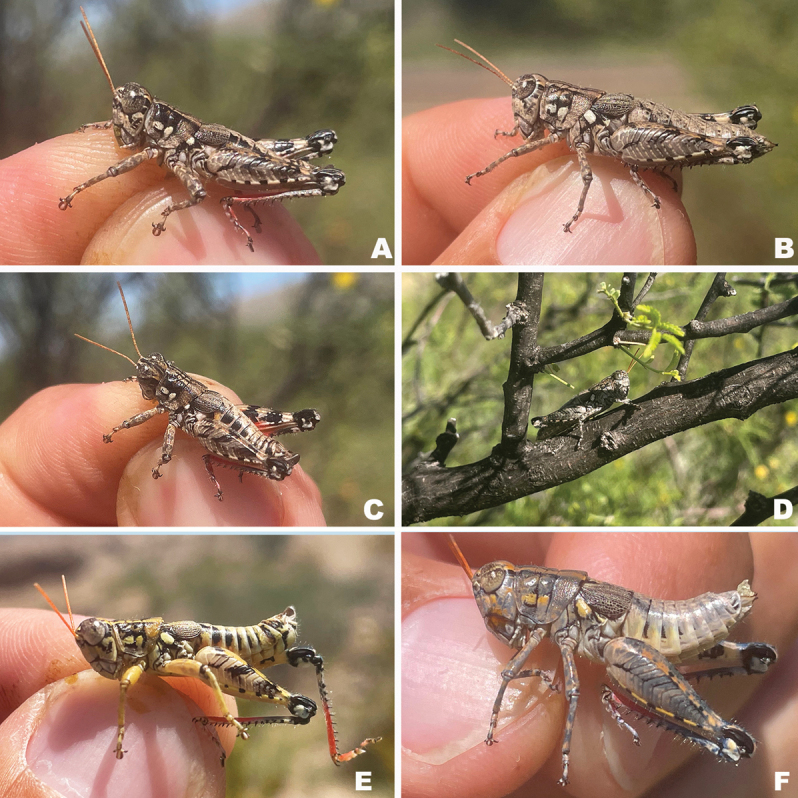
*Agroecotettixturneri* from Brewster Co., Texas **A** male **B** female **C** male **D** female on *Vachellia*.

#### Etymology.

The species name *kahloae* patronym honoring Frida Kahlo (1907–1954), the iconic Mexican painter known for her vivid deeply personal and symbolic artwork. Her enduring connection to Mexican culture makes her an apt figure to be commemorated through this species, which is endemic to Mexico. In naming a species in her honor I celebrate her artistic legacy and underscore the importance of preserving the biodiversity of her homeland.

#### Suggested common name.

Kahlo’s aridland scrub jumper.

## ﻿Discussion

The discovery of sixteen new species of *Agroecotettix*, predominantly comprising endemics of the Chihuahuan Desert, Sierra Madre Occidental, and the South Texas Plains, presents a significant advancement in our knowledge of desert biodiversity and the ecological complexity of this unique region. However, without population level genetic data, it is challenging to definitively pinpoint the factors that led to the diversification and biogeographic patterns observed here. Evolution of other brachypterous groups of North American melanoplines were influenced by Pleistocene glacial cycles that impacted river flow, mountain ecosystems, and the isolation of islands/sand ridges, which resulted in population cycles of contraction, isolation, divergence, expansion, and secondary contact processes. (Knowles 2007; Woller 2017; Huang et al. 2020). Indeed, this may be case for *Agroecotettix* as well.

The Chihuahuan Desert (Fig. 25) stands out as the center of diversity for *Agroecotettix*, with eleven of the nineteen species inhabiting this area, nine of which are endemic. This desert, known for its biological diversity and vast expanse, hosts a rich variety of plant and animal life, including numerous endemics specially adapted to its arid conditions (Medellin-Leal 1982; Toledo and Ordóñez 1993; Villarreal-Quintanilla et al. 2017; Scheinvar et al. 2020).

Today, this desert is inhabited by a myriad of specialized plants and animals, including cacti, yuccas, reptiles, mammals, and a diverse array of invertebrates. However, during the Pleistocene, especially the Late Wisconsin (27,000–11,000 yr B.P.), the Chihuahuan Desert was a very different place. During that time, the area around Big Bend and the New Mexico/Arizona borderline were dominated by a woodland of paper-shell pinyon and juniper, and the Mapimian region vegetation assemblages were dominated by coniferous/juniper forest. The microphyllous Desertic Brushwood system that *Agroecotettix* is today associated with such as *Acacia* and sotol (*Dasylirion* spp.) as well as other characteristic Chihuahuan desert elements such as lechuguilla (*Agavelechuguilla*), and prickly pears (*Opuntia* spp.) was much rarer (Betancourt et al. 1990; Scheinvar et al. 2020). Establishment of Chihuahuan desert scrub as a dominant element was not recorded until 8,000–9,000 yr B.P. (Betancourt et al. 1990; Holmgren et al. 2003).

Similarly, Toomey et al. (1993) described the environment of the Edwards Plateau (Fig. 25) during the late Pleistocene (ca 20–14,000 yr B.P.) as having much of the uplands covered in a deep reddish-clay soil and open mixed tall and short grass savanna. Drying conditions during the Holocene (10,500–2,500 yr B.P.) resulted in diminished vegetation cover which caused the gradual degradation of soil mantles and a shift to short grasses and scrub plant communities (Toomey et al. 1993. The Edwards Plateau and surrounding areas, in contrast to the Chihuahuan Desert, host the most widespread *Agroecotettix*, namely, *A.aristus*. This distribution suggests that the species and genus might have originated in the south and spread to their current range during a more recent arid period.

Specimens from my fieldwork in the United States have recently been sent for sequencing, but there are still large collecting gaps in Mexico, and little genetic data currently available for specimens there. It is hoped that this article will stir interest in searching for more *Agroecotettix* species to help tell the story of the biogeographic history of this interesting region.

## Supplementary Material

XML Treatment for Agroecotettix

XML Treatment for Agroecotettix modestus

XML Treatment for Agroecotettix silverheelsi

XML Treatment for Agroecotettix aristus

XML Treatment for Agroecotettix xiphophorus

XML Treatment for Agroecotettix glochinos

XML Treatment for Agroecotettix texmex

XML Treatment for Agroecotettix cumbres

XML Treatment for Agroecotettix crypsidomus

XML Treatment for Agroecotettix burtoni

XML Treatment for Agroecotettix moorei

XML Treatment for Agroecotettix chiantiensis

XML Treatment for Agroecotettix dorni

XML Treatment for Agroecotettix chisosensis

XML Treatment for Agroecotettix turneri

XML Treatment for Agroecotettix quitmanensis

XML Treatment for Agroecotettix vaquero

XML Treatment for Agroecotettix forcipatus

XML Treatment for Agroecotettix idic

XML Treatment for Agroecotettix kahloae

## ﻿Additional information

### Author ORCIDs

JoVonn G. Hill https://orcid.org/0000-0002-1892-7117

### Data availability

All of the data that support the findings of this study are available in the main text.

## References

[B1] Barrientos-Lozano L Rocha-Sánchez AY Horta-Vega J (2013a) Two new species of *Melanoplus* Stål, 1873 (Orthoptera: Acrididae) from northeastern Mexico. Zootaxa 3669: 261–286. 10.11646/zootaxa.3669.3.4 26312342

[B2] Barrientos-Lozano L Rocha-Sánchez AY Buzzetti FM Méndez-Gómez BR Horta-Vega JV (2013b) In Saltamontes y esperanzas del noreste de México (Insecta: Orthoptera). Guía ilustrada. Miguel Ángel Porrúa, Ciudad de México, 388 pp.

[B3] Betancourt JL Van Devender TR Martin PS Paul S (1990) Packrat middens: the last 40,000 years of biotic change. University of Arizona Press, Tucson, AZ, 478 pp. 10.1126/science.250.4983.1021-a 17746928

[B4] Bruner L (1908) Orthoptera. The Acrididae. Biologia Centrali-Americana 2: 249–342.

[B5] Carbonell CS (2007) The genus *Zoniopoda* Stål 1873 (Acridoidea, Romaleidae, Romaleinae). Journal of Orthoptera Research 16(1): 1–33. 10.1665/1082-6467(2007)16[1:TGZSAR]2.0.CO;2

[B6] Cigliano MM Braun H Eades DE Otte D. (2024) Orthoptera Species File. http://orthoptera.speciesfile.org/ [Accessed 22 July 2024]

[B7] Cohn TJ (1955) UMMZI-FN358. [Accessed online at] https://quod.lib.umich.edu/cgi/i/image/image-idx?id=S-INSECT1IC-X-401%5DUMMZI-FN358_001 [Accessed 14 May 2024]

[B8] Cohn TJ (1956) UMMZ-FN294. [Accessed online at] https://quod.lib.umich.edu/cgi/i/image/image-idx?id=S-INSECT1IC-X-348%5DUMMZI-FN294_001 [accessed 15 May 2024]

[B9] Cohn TJ (1959) UMMZ-FN298. [Accessed online at] https://quod.lib.umich.edu/cgi/i/image/image-idx?id=S-INSECT1IC-X-352%5DUMMZI-FN298_001 [accessed 15 May 2024]

[B10] Cohn TJ (1964) UMMZ-FN300. [Accessed online at] https://quod.lib.umich.edu/cgi/i/image/image-idx?id=S-INSECT1IC-X-354%5DUMMZI-FN300_001 [accessed 15 May 2024]

[B11] Cohn TJ (1965) UMMZ-FN301. [Accessed online at] https://quod.lib.umich.edu/cgi/i/image/image-idx?id=S-INSECT1IC-X-355%5DUMMZI-FN301_001 [accessed 15 May 2024]

[B12] Eades DC (2000) Evolutionary relationships of phallic structures of Acridomorpha (Orthoptera). Journal of Orthoptera Research 9: 181–210. 10.2307/3503648

[B13] Fontana P Buzzetti FM Mariño-Pérez R (2008) Chapulines, Langostas, Grillos y Esperanzas de México. Guía fotográfica - Grasshoppers, Locusts, Crickets & Katydids of Mexico. Photographic guide. World Biodiversity Association, Verona, Italy, 272 pp.

[B14] Gurney AB Brooks AR (1959) Grasshoppers of the *Mexicanus* Group, Genus *Melanoplus* (Orthoptera: Acrididae). Proceedings of the United States National Museum 110: 1–93. 10.5479/si.00963801.110-3416.1

[B15] Hebard M (1922) New genera and species of Melanopli found within the United States and Canada (Orthoptera: Acrididae): Part IV. Transactions of the American Entomological Society 48: 49–66.

[B16] Hill JG (2015) Revision and Biogeography of the *Melanoplusscudderi* Species group (Orthoptera: Acrididae: Melanoplinae) with a Description of 21 New Species and Establishment of the Carnegiei and Davisi Species Groups. Transactions of the American Entomological Society 141: 252–350. 10.3157/061.141.0201

[B17] Hill JG (2023) Diversification deep in the heart of Texas: seven new grasshopper species and establishment of the *Melanoplusdiscolor* species group (Orthoptera: Acrididae: Melanoplinae). ZooKeys 1165: 101–136. 10.3897/zookeys.1165.104047 37304569 PMC10251248

[B18] Holmgren CA Peñalba MC Rylander KA Betancourt JL (2003) A 16,000 14C yr B.P packrat midden series from the USA-Mexico borderlands. Quaternary Research 60: 319–329. 10.1016/j.yqres.2003.08.001

[B19] Huang JP Hill JG Ortego J Knowles LL (2020) Paraphyletic species no more—genomic data resolve Pleistocene radiation and validate morphological species of the *Melanoplusscudderi* species complex (Insecta: Orthoptera). Systematic Entomology 45(3): 594–605. 10.1111/syen.12415

[B20] Hubbell TH (1932) A revision of the *Puer* Group of the North American genus *Melanoplus*, with remarks on the taxonomic value of the concealed male genitalia in the Cyrtacanthacrinae (Orthoptera, Acrididae). University of Michigan Museum of Zoology Miscellaneous Publication 23, 64 pp.

[B21] Knowles LL (2007) Tests of Pleistocene speciation in montane Grasshoppers (Genus *Melanoplus*) from the sky islands of western North America. Evolution 54: 1337–1348. 10.1111/j.0014-3820.2000.tb00566.x 11005300

[B22] Medellin-Leal F (1982) The Chihuahuan Desert. In: Bender GL (Ed.) Reference handbook on the Deserts of North America.Greenwood Press, Westport, CT, 321–381. [594 pp]

[B23] Otte D (2012) Eighty new *Melanoplus* species from the United States (Acrididae: Melanoplinae). Transactions of the American Entomological Society 138: 73–167. 10.3157/061.138.0103

[B24] Otte D (2019) Revision of the genus *Paraidemona* Bruner Von Wattenwyl 1893 (Acrididae: Melanoplinae). Transactions of the American Entomological Society 145: 435–535. 10.3157/061.145.0308

[B25] Scheinvar E Gámez N Moreno-Letelier A Aguirre E Eguiarte LE (2020) Phylogeography of the Chihuahuan Desert: Diversification and Evolution Over the Pleistocene. In: Mandujano M Pisanty I Eguiarte LE (Eds) Plant Diversity and Ecology in the Chihuahuan Desert: Emphasis on the Cuatro Ciénegas Basin.Springer, Cham, 19–44. 10.1007/978-3-030-44963-6_2

[B26] Toledo VM Ordóñez MJ (1993) The biodiversity scenario of Mexico: a review of terrestrial habitats. In: Ramamoorthy TP Bye RA Lot A Fa J (Eds) Biological diversity of Mexico: origins and distribution.Oxford University Press, New York, NY, 757–777.

[B27] Toomey III RS Blum MD Valastro Jr S (1993) Late Quaternary climates and environments of the Edwards Plateau, Texas. Global and Planetary Change 7: 299–320. 10.2993/0278-0771-33.2.170

[B28] Villarreal-Quintanilla JA Bartolomé-Hernández JA Estrada-Castillón E Ramírez-Rodríguez H Martínez-Amador SJ (2017) El elemento endémico de la flora vascular del Desierto Chihuahuense. Acta Botánica Mexicana 118: 65–96. 10.21829/abm118.2017.1201

[B29] Woller D (2017) Xerophilic Flightless Grasshoppers (Orthoptera: Acrididae: Melanoplinae: *Melanoplus*: The Puer Group) of the Southeastern U.S.A.: An Evolutionary History. Texas A&M Dissertation, 341 pp.

